# Potential therapeutic effects of *cyanidin-3-O-glucoside* on rheumatoid arthritis by relieving inhibition of CD38+ NK cells on Treg cell differentiation

**DOI:** 10.1186/s13075-019-2001-0

**Published:** 2019-10-28

**Authors:** Hongxing Wang, Shutong Li, Guoqing Zhang, Hui Wu, Xiaotian Chang

**Affiliations:** 1 Shandong Provincial Qianfoshan Hospital, Shandong University, Jingshi Road 16766, Jinan, 250014 Shandong People’s Republic of China; 2 Medical Research Center of The Affiliated Hospital of Qingdao University, Wutaishan Road 1677, Qingdao, 266000 Shandong People’s Republic of China; 3 Qingdao Engineering Technology Center For Major Disease Marker, Wutaishan Road 1677, Qingdao, 266000 Shandong People’s Republic of China

**Keywords:** CD38, Cyanidin-3-O-glucoside (C3G), IFN-γ, IL-10, CD38+ NK cell, Mononuclear cells (MNCs), Natural killer group 2D (NKG2D), Rheumatoid arthritis (RA), Sirtuin 6 (Sirt6), Treg cell, TNF-α

## Abstract

**Background:**

CD38+ NK cells are overabundant in rheumatoid arthritis (RA). Cyanidin-3-O-glucoside (C3G) is an inhibitor of CD38. This study investigated the pathogenic role of CD38+ NK cells and the effect of C3G on RA.

**Methods:**

Rats with bovine type II collagen-induced arthritis (CIA) were injected with C3G. RA synovial fibroblasts (RASFs) or mononuclear cells (MNCs) were cultured with C3G. MNCs were also cocultured with CD38+ NK cells following C3G pretreatment.

**Results:**

C3G injection significantly alleviated CIA. C3G also significantly increased the level of interleukin (IL)-10 and the regulatory T (Treg) cell proportion, and it decreased the interleukin (IL)-6 and interferon (IFN)-γ levels and CD38+ NK cell proportion in rat peripheral blood and synovial fluid. Additionally, C3G significantly increased RASF apoptosis and decreased RASF proliferation and IL-6 production in the culture medium. Furthermore, C3G stimulated MNCs to increase IL-2 and IL-10 production and the Treg cell proportion, and it caused MNCs to decrease IL-6 and IFN-γ production and the CD38+ NK cell proportion. Although CD38+ NK cells significantly decreased the Treg cell proportion and IL-10 level in MNCs, CD38+ NK cells that had been pretreated with C3G increased the proportion of Treg cells and IL-10 levels and decreased the IL-6 and IFN-γ levels in the coculture. In CD38+ NK cells, C3G significantly increased Sirtuin 6 (Sirt6) expression and the tumor necrosis factor (TNF)-α level, and it decreased natural killer group 2D (NKG2D) expression and the IFN-γ level. However, when CD38+ NK cells were treated with Sirt6 siRNA, C3G did not change the NKG2D expression, the TNF-α level sharply decreased, and the IFN-γ level increased. When MNCs were cocultured with C3G-pretreated CD38+ NK cells in the presence of TNF-α and an anti-IFN-γ antibody, the IL-10+ Treg cell proportion significantly increased. When MNCs were cocultured with C3G-pretreated CD38+ NK cells in the presence of IFN-γ and an anti-TNF-α antibody, the IL-10+ Treg cell proportion sharply decreased. When CIA rats were injected with both C3G and the Sirt6 inhibitor OSS_128167, the rats exhibited joint inflammation and a low Treg cell proportion, but the CD38+ NK proportion was still low.

**Conclusion:**

C3G has therapeutic effects on CIA and RA. C3G decreased the proportion of CD38+ cells, RASF proliferation, and proinflammatory cytokine secretion, and it increased the Treg cell proportion. C3G also elevated Sirt6 expression to suppress NKG2D expression, increase TNF-α secretion, and decrease IFN-γ secretion in CD38+ NK cells, which stimulates MNCs to differentiate into Treg cells. This study also demonstrates that the inhibition of Treg cell differentiation in MNCs by CD38+ NK cells is a potential cause of the immune imbalance in RA and CIA.

## Background

Rheumatoid arthritis (RA) is a common autoimmune disease [1–3]. At present, the pathological mechanisms of RA are not fully understood, and the drugs available for treating this disease are limited. CD38 is a glycoprotein on the cell membrane that functions as a cyclic ADP-ribose hydrolase (cADPRH). CD38 catalyzes the conversion of nicotinamide adenine dinucleotide (Coenzyme I, NAD+) to cyclic ADP-ribose (cADPR) to regulate calcium ion (Ca^2+^) homeostasis. Through transcriptome analysis, we found that CD38 was highly expressed in RA synovial tissue. Postigo et al. found that knockout of CD38 in mice significantly alleviated the occurrence and development of collagen-induced arthritis (CIA) [4]. We also detected a high proportion of CD38+ CD56+ cells in the peripheral blood of RA patients [5]. CD56 is an important marker of natural killer (NK) cells [6]. Studies have shown that NK cells contribute to the progression of RA by regulating the secretion of tumor necrosis factor (TNF)-α, interferon (IFN)-γ, and other cytokines to modulate the functions of immune cells, such as B cells, T cells, macrophages, and fibroblasts [7–14]. Thus, we hypothesized that CD38 and CD38+ NK cells play important roles in RA and might be therapeutic targets [5, 15].

Cyanidin-3-O-glucoside, which is usually found in the form of chlorinated cyanidin-3-O-glucoside (kuromaninchloride, cyanidin-3-O-glucoside chloride, 1-benzopyrylium, 5,7-dihydroxy-2-(3,4-dihydroxyphenyl)-3-(β--D-glucopyranosyloxy)-chloride), is referred to as C3G. The molecular formula of C3G is C_21_H_21_ClO_11_. The structural core of C3G is a 2-phenylbenzopyran cation, which belongs to a family of flavonoid compounds. C3G has antitumor, anti-inflammatory, and antioxidant effects [16–21]. Studies also found that C3G is an inhibitor of CD38 and competitively inhibits the function of CD38 by binding to the active site [22]. This binding prevents CD38 from catalyzing the synthesis of cADPR and leads to accumulation of intracellular NAD (+) in dendritic cells, HL-60 cells, and chronic lymphocytic leukemia [23–25]. Therefore, we postulated that C3G might be used to treat RA by inhibiting CD38 activity. Although increasing evidence shows that C3G, which is naturally derived from many plants, may provide protection against neurodegenerative diseases [26–29], no other molecular target except CD38 has been reported.

We aimed to determine the pathogenic role of CD38 and the effect of C3G on RA in this study, which may provide a basis for developing C3G as a therapeutic agent for RA. In the present study, C3G was used to treat CIA model rats, and it was cultured with RA synovial fibroblast (RASF)-like cells, cultured mononuclear cells (MNCs), and CD38+ NK cells derived from RA peripheral blood or synovial fluid. The changes in lymphocyte subsets and proinflammatory cytokine levels were measured. Additionally, CD38+ NK cells with C3G treatment were cocultured with MNCs depleted of CD38+ NK, and the mechanism of MNC differentiation into T regulatory (Treg) cells was investigated. Sirtuin 6 (Sirt6) is a member of the sirtuin protein family and alleviates inflammatory responses in CIA mice [30, 31]. This study also explored the effects of C3G on Sirt6 expression and its downstream pathways in CD38+ NK cells.

## Methods

### Tissue collection

Human blood (*n* = 30, 26 females, 21–81 years old, mean age of 56) and synovial fluid (*n* = 20, 15 females, 25–78 years old, mean age of 58) were collected from patients with RA. RA synovial membrane tissue was collected from the patients (*n* = 7, 6 females, 23–77 years old, mean age of 60) during knee joint arthroscopic synovectomy. The diagnosis conformed to the revised criteria of the American College of Rheumatology. All patients provided written informed consent for the study. The study protocol was approved by the Medical Ethics Committee of The Affiliated Hospital of Qingdao University at Qingdao, Shandong of China (Approval number: 20181201, China). The detailed characteristics of RA patients are shown in Additional file 6: Table S1.

### Preparation of an animal model with CIA

Six-week-old Sprague Dawley (SD) rats (*n* = 72, male) were purchased from the Shandong Laboratory Animal Center (China). The breeding and care of the experimental animals were carried out in accordance with the Helsinki Convention on Animal Protection and the Regulations of the People’s Republic of China on the Administration of Experimental Animals. The animals were randomly divided into a normal control (NC) group (*n* = 24) and a CIA control group (*n* = 48). Bovine type II collagen (Chondrex, USA) was mixed with complete Freund’s adjuvant (Sigma-Aldrich, USA), and the initial immunization was performed by intracutaneous injection at the tail root. One week later, bovine type II collagen was mixed with incomplete Freund’s adjuvant (Sigma-Aldrich), and the booster immunization was performed by intracutaneous injection at the tail root. The NC group was injected with an equal volume of phosphate-buffered saline (PBS).

### Treatment of CIA rats with cyanidin-3-O-glucoside chloride (C3G)

C3G, also called kuromanin chloride (Solarbio, China), was dissolved in PBS. Ten days after the secondary immunization, the CIA rats were randomly divided into a CIA control group (*n* = 24) and a C3G treatment group (*n* = 24). The C3G treatment group was injected with the C3G solution (25 mg/kg) via the tail vein. The injection was carried out twice per week for six consecutive administrations. The NC and CIA control groups were simultaneously injected with the same volume of PBS. The inflammation curve of joint swelling degree over time was plotted.

### Histopathological examinations

The animals were sacrificed 20 days after the first C3G injection. The joint tissue located 0.5 cm around the rat knee joints was collected, fixed in 4% paraformaldehyde, and embedded in paraffin. Pathological changes in the joint tissue were examined using hematoxylin-eosin (HE) staining. Disease scores were calculated according to clinical and histologic evidence as follows: 0 = no inflammation (normal joint), 1 = local swelling and/or erythema without histologic lesions, 2 = swelling of the entire paw and/or ankylosis without histologic lesions, 3 = limb deformities with reversible histologic lesions, 4 = limb deformities accompanied by permanent histologic lesions such as bone or cartilage erosions [32, 33].

### Detection of lymphocyte subtypes in rats

Animals were anesthetized by intraperitoneal injection of 3% sodium pentobarbital. Blood samples were collected from the inferior vena cava. The ankle joints of rats were opened, and the joint cavities were flushed with 0.5 mL of PBS. The joint cavity lavage fluid was collected as a synovial fluid sample. The peripheral blood and synovial fluid samples were treated with red blood cell lysis buffer (BioLegend, USA). The samples were incubated with the following antibodies at 4 °C for 30 min in the dark. Fluorescein isothiocyanate (FITC)-conjugated anti-rat CD3 (BioLegend) was used to detect CD3+ T cells; allophycocyanin (APC)-conjugated anti-rat CD3 and FITC anti-rat CD4 (BioLegend) were used for CD3+ CD4+ T cells; APC anti-rat CD3 (BioLegend) and peridinin chlorophyll protein complex (PerCP)-conjugated anti-rat CD8a (BioLegend) were used for CD3+ CD8+ T cells; APC anti-rat CD4 (BioLegend), FITC anti-rat CD25 (BioLegend), and phycoerythrin (PE)-conjugated anti-rat Foxp3 (BioLegend) were used for Treg cells; APC anti-rat CD3 (BioLegend) and PE anti-rat CD45RA (BioLegend) were used for B cells [34]; APC anti-rat CD3 and FITC anti-rat CD161 (BioLegend) were used for CD3− CD161+ NK cells; and APC anti-rat CD3, PE anti-rat CD38 (BioLegend), and FITC anti-rat CD161 (BioLegend) were used for CD38+ CD3− CD161+ NK cells (CD38+ NK cells). Lymphocyte subtypes were detected with flow cytometry (American ACEA BIO, Novo Cyte D2040R).

### Detection of cytokine concentrations in rats

Cytokine expression in the peripheral blood and synovial fluid was detected by flow cytometry using a rat Th1/Th2 Cytokine Assay kit (BioLegend). The magnetic beads, assay buffer, antibody, and standard or tested sample were incubated together for 3 h at room temperature. The concentrations of granulocyte-macrophage colony-stimulating factor (GM-CSF), IFN-γ, interleukin (IL)-2, IL-4, IL-5, IL-10, IL-6, IL-13, and TNF-α in rat serum and synovial fluid were measured with flow cytometry.

### RASF culture and C3G treatment

RA synovial tissue (*n* = 7, from patient no. 28 to no. 34) was aseptically homogenized, and DMEM containing 4% type II collagenase (Solarbio, China) was added to the samples before incubation in a 5% CO_2_ incubator at 37 °C for 4 h. The cell suspension was filtered through a 70-μm cell strainer and centrifuged. The cells were resuspended in DMEM containing 10% FBS and cultured at 37 °C in a 5% CO_2_ incubator to obtain primary RASFs. Cells passaged for 3–8 generations were used in subsequent experiments. C3G solution was added to the RASF culture at final concentrations of 25, 50, or 100 μM, and the cells were incubated.

### Detection of RASF cell proliferation

Following C3G treatment, the suspended RASF cells were washed away with PBS, and CCK-8 solution (Dojindo, Japan) was added to the culture. The OD_450_ value was measured with a spectrophotometer (BioTek, USA).

### Detection of RASF apoptosis

Following C3G treatment, trypsin was added to the RASF culture, and the cells were collected by centrifugation. The binding buffer was added to resuspend the cell pellet, and FITC-conjugated anti-Annexin V (BioLegend) antibody and PI-conjugated antibody (BioLegend) were then added to the suspended RASF cells in dark for 15 min. Apoptosis was measured by flow cytometry.

### Detection of human cytokine concentrations

A Human Th1/Th2 Subgroup Detection Kit (CellGene, China) was used to detect various cytokines in human bodily fluids and culture supernatant. RASFs were cultured in C3G, and the supernatant was collected. The mixed capture microsphere solution, fluorescent reagent, and sample to be tested were incubated together for 2.5 h at room temperature. The mixture was centrifuged and resuspended in PBS. The concentrations of IL-2, IL-4, IL-6, IL-10, TNF-α, and IFN-γ were measured by flow cytometry.

### Preparation of peripheral blood mononuclear cells

Peripheral blood of RA patients (*n* = 18, from patient no. 1 to no. 18) with anticoagulants was collected and centrifuged at 500×*g* for 10 min and resuspended in PBS. The suspension was added to the separation solution (Haoyanghuake Biology, China) and centrifuged at 800×*g* for 20 min. The white ring of the MNC layer was collected, washed, and resuspended in PBS. The cells were resuspended with RPM 1640 containing 10% FBS.

### Preparation of synovial fluid mononuclear cells (SMNCs)

The RA synovial fluid samples (*n* = 18, from patient no. 14 to no. 31) were collected and pretreated with hyaluronidase (10 U/mL) (Solarbio) at 37 °C for 30 min. MNCs in the synovial fluid were separated by density gradient centrifugation with lymphocyte separation solution (Haoyanghuake Biology, China). Following centrifugation, MNCs from RA synovial fluids were resuspended with RPM 1640 medium containing 10% FBS.

### Treatment of MNCs with C3G

The synovial MNCs or blood MNCs were seeded at a density of 2 × 10^5^ cells/mL into a 96-well U-bottom plate (Corning Costar) and cultured in serum-free medium. C3G (or an equal volume PBS) was added to the final concentration of 50 μM, and the cells were incubated at 37 °C for 48 h in 5% CO_2_. The cell suspensions were collected by centrifugation. The Human Th1/Th2 Subgroup Detection kit was applied to detect levels of various cytokines in the suspensions, as described above.

### Detection of human lymphocyte subtypes

Cultured MNCs were collected from each well and centrifuged. The cell pellet was resuspended in PBS. The sample to be tested was incubated with the following antibodies at 4 °C for 30 min. CD45+ lymphocytes, CD3+ T cells, CD3+ CD4+ T cells, and CD3+ CD8+ T cells were detected with the FITC anti-CD3/PE anti-CD8/PerCP anti-CD45/APC anti-CD4 detection kit (ACEA Biosciences, China). CD3− CD56+ CD16+ NK cells and CD3− CD19+ B cells were detected using a FITC anti-CD3/PE anti-CD16+ CD56/PerCP anti-CD45/APC anti-CD19 detection kit (ACEA biosciences). CD4+ CD25+ Foxp3+ Treg cells were detected with FITC anti-human-CD25 (BioLegend), PerCP/Cy5.5 anti-human-CD4 (BioLegend), and PE anti-human-Foxp3 (BioLegend). CD38+ CD3− CD56+ NK cells were detected with PE anti-CD38 (BioLegend), FITC anti-CD3 (BioLegend), and APC anti-CD56 (BioLegend). Lymphocyte subtypes within MNCs were measured with flow cytometry.

### Treg cell isolation and sorting

MNCs were obtained from the peripheral blood of RA patients (*n* = 9, from patient no. 13 to no. 21). The MNCs were incubated with FITC anti-human-CD25 (BioLegend), PerCP/Cy5.5 anti-human-CD4 (BioLegend), and FE anti-human-CD127 (BioLegend) antibodies for 30 min at room temperature. The cells were collected by centrifugation and resuspended in PBS. The labeled cells were sorted using a BD FACS ARIA II with BD FACS Diva V6 software (BD Biosciences, USA). The Treg cells were analyzed for purity. This method is common for Treg cell preparation [35].

### Treatment of Treg cells with C3G and identification of IL-10+ Treg cells

Treg cells were seeded in 96-well U-bottom plates. C3G at concentrations of 25, 50, or 100 μM (or an equal volume of PBS) was added, and the cells were incubated in 5% CO_2_ at 37 °C for 48 h. The Treg cells were collected by centrifugation and resuspended in PBS. FITC anti-human-CD25 and PerCP anti-human-CD4 antibodies were added, and the cultured cells were incubated at 4 °C for 30 min. The cells were fixed with fixation buffer (BioLegend) and incubated for 20 min at room temperature. Following centrifugation, the cells were resuspended with cell staining buffer (BioLegend) and centrifuged. The cells were then resuspended with a permeabilization wash buffer (BioLegend) and centrifuged. PE anti-human IL-10 was added (BioLegend), and the mixture was incubated at room temperature for 20 min. After centrifugation, the stained cells were resuspended in cell staining buffer (BioLegend) and analyzed by flow cytometry.

### Isolation and sorting of CD38+ NK cells and MNCs without CD38+ NK cells

MNCs were obtained from the peripheral blood of RA patients (*n* = 9, from patient no. 22 to no. 30). The MNCs were incubated with FITC-conjugated anti-human CD3, PE-conjugated anti-human CD38, and APC-conjugated anti-human CD56 for 30 min at room temperature. The cells were centrifuged and resuspended in PBS for sorting. The labeled cells were sorted using a BD FACS ARIA II with BD FACS Diva V6 software (BD Biosciences). CD3− CD38+ CD56+ cells and CD38+ NK-depleted MNCs were separated. After sorting, the purity of the cell fractions was measured on a BD FACS ARIA (BD Immunocytometry Systems, Belgium) equipped with a 70-μm nozzle. Sorted cells were collected in RPMI 1640 medium containing 10% FBS. This experiment followed Meggyes’s design [36].

### CD38+ NK cell cytotoxicity assays

CD38+ NK cells and autologous Treg cells were isolated from the peripheral blood of RA patients using flow cytometry-based methods, as described above. CD38+ NK cells were incubated in 50 μM C3G or an equal volume of PBS at 37 °C for 24 h. Isolated Treg cells were labeled with 5 μM carboxy fluorescein diacetate succinimidyl ester (CFSE; BioLegend) for 10 min at 37 °C. The pretreated CD38+ NK cells and Treg cells were washed and seeded at an effector-to-target cell (E:T) ratio of 20:1 in a 96-well U-bottom plate for 4 h at 37 °C. 7-Aminoactinomycin D (7-AAD; 5 μg/ml; BioLegend) was added to the culture, and the mixture was incubated for 20 min at 4 °C. Cell death was measured using flow cytometry. The percentage of specific lysis was calculated based on the following formula: 100 × (CFSE+ 7-AAD+ Treg cells/total number of CFSE+ Treg cells). The flow cytometry-based cytotoxicity assay for CD38+ NK cells against Treg cells was designed following a previously reported method [37, 38].

### Coculture of CD38+ NK cells and CD38+ NK-depleted MNCs

CD38+ NK cells (*n* = 9) from the peripheral blood of RA patients were incubated in media containing 50 μM C3G or an equal volume of PBS at 37 °C for 24 h. The pretreated cells were washed twice, collected in serum-free RPMI 1640, and then moved to the upper chamber of a 0.4-μm transwell apparatus (Corning Costar, USA). MNCs depleted of CD38+ NK cells from the same source were seeded into the lower chamber of the transwell apparatus. TNF-α (10 ng/mL, Sigma), IFN-γ (5 ng/mL, Sigma), anti-TNF-α antibody (10 ng/mL, Cell Signaling, USA), or anti-IFN-γ antibody (5 ng/mL, Cell Signaling) were added or not in the culture medium. The MNCs depleted of CD38+ NK cells were treated with 50 μM C3G as a control. The culture was incubated at 37 °C and 5% CO_2_ for 48 h. Flow cytometry was used to analyze the proportion of IL-10+ Treg cells in the MNCs.

### Detection of Treg cell apoptosis

MNCs or CD38+ NK cells were incubated in media containing 50 μM C3G or an equal volume of PBS at 37 °C for 24 h. CD38+ NK cells were then washed twice and cocultured with CD38+ NK-depleted MNCs in a transwell apparatus at 37 °C and 5% CO_2_ for 48 h. The MNCs or CD38+ NK-depleted MNCs were incubated with PerCP anti-CD4 antibody (BioLegend) and APC anti-CD25 antibody (BioLegend) for 30 min. After washing with PBS and centrifugation, binding buffer was added to resuspend the cell pellet. FITC anti-Annexin V antibody (BioLegend) was added to the cells, and they were incubated in the dark for 15 min. The percentage of annexin V+ CD4+ CD25+ Treg cells in the CD4+ CD25 Tregs was quantified by flow cytometry using routine methods.

### Detecting Sirt6 and NKG2D (natural killer group 2, member D) expression in CD38+ NK cells using real-time polymerase chain reaction (PCR)

CD38+ NK cells were obtained by sorting the peripheral blood of RA patients (*n* = 9) and were stimulated with PBS or C3G at 25, 50, or 100 μM for 48 h. The cells were collected, and RNA was extracted from the cells and then transcribed into cDNA using a TOYOBO reverse transcription kit (Japan). A StepOnePlus™ Real-Time PCR System (Thermo Fisher Scientific, USA) was used to detect Sirt6 and NKG2D mRNA levels. The sequence of PCR primers are as follows: Sirt6 forward 5′ TGGCAGTCTTGTGTGTGTTGT 3′ and reverse 5′ CGCTCAAAGGGTGTCGAA 3′; NKG2D forward 5′ ACTGTGGCCCATGTCCTTAAA 3′ and reverse 5′ GGTTGGGTGAATGGAG 3′.

### Transfection of CD38+ NK cells with Sirt6 siRNA

The siRNA sequence targeting Sirt6 was as follows:

5′ CCGGCTCTGCACCGTGGCTAA 3′. The cultured CD38+ NK cells were transfected with Sirt6 siRNA for 24 h using HiPerFect transfection reagent (Qiagen). Parallel experiments were conducted with the Allstars nontargeting siRNA provided with the kit. The cells were collected by centrifugation and then cultured for 48 h with 50 μM C3G. The supernatant of the cell culture was collected, and the cytokine concentrations were detected by flow cytometry. The cells were collected simultaneously. Total RNA was routinely extracted, and Sirt6 and NKG2D mRNA levels were detected using real-time PCR.

### Western blotting

The cultured cells were treated with tissue lysate buffer (Beyotime, China). After centrifugation at 10,000×*g* and 4 °C for 20 min, the supernatant was collected, separated on 10–12% SDS-PAGE gels, and transferred to polyvinylidene fluoride (PVDF) membranes (Millipore, USA). Anti-Sirt6 antibody (Abcam, USA) was used to detect the corresponding target protein expression. After incubation with HRP-conjugated secondary antibody, the immune signals were detected with a Western chemiluminescent HRP substrate (Millipore).GAPDH was used as an internal reference to normalize the expression levels of the target proteins. A commercial antibody against GAPDH was obtained from Abcam.

### Treatment of CIA rats with Sirt6 inhibitors

The Sirt6 inhibitor OSS-128167 (Selleck Chemical, USA) was dissolved to a final concentration of 12 mg/mL in a solution containing 2% DMSO, 40% PEG 300, and 2% Tween-80. CIA rats were prepared and randomly divided into four groups including the C3G treatment group (*n* = 9), C3G combined with Sirt6 inhibitor treatment group (*n* = 9), Sirt6 inhibitor treatment group (*n* = 9), and CIA control group (*n* = 9). The Sirt6 inhibitor solution (100 mg/kg) or an equal volume of the carrier solution or C3G (25 mg/kg) was injected into the tail vein of the corresponding CIA rats. The rats were injected twice weekly for a total of six times. Peripheral blood and synovial fluid samples were collected. The proportions of Treg cells and CD38+ NK cells as well as the levels of TNF-α, IFN-γ, IL-6, and IL-10 were measured by flow cytometry.

### Statistical analysis

Normal and variance homogeneity tests were performed using SPSS 17.0 software (IBM, USA). One-way ANOVA was used for multigroup significance tests. The least significant difference (LSD) method was used for pairwise comparisons. The test standard was *α* = 0.05, and *p* < 0.05 indicated statistical significance.

## Results

### Effects of C3G on CIA rats

The CIA rats were treated with C3G. Compared with the CIA model group, C3G-treated rats had alleviated redness and swelling of the toes and partially restored joint mobility (Additional file 1: Figure S1A). Histochemical staining showed few cartilage changes, synovial hyperplasia, and a small amount of inflammatory cell infiltration in the C3G treatment group (Additional file 1: Figure S1B). The inflammation curve showed that approximately 16 days after treatment, the toe swelling of the CIA model group peaked (*p* < 0.0001). The toe swelling of the CIA model group on day 20 after treatment began was 6.71 ± 3.40 mm, while the toe swelling of the C3G treatment group was 2.78 ± 2.70 mm (Additional file 1: Figure S1C). The disease score of the CIA model group was 2.42 ± 0.98, while the disease score of the C3G treatment group was 1.51 ± 0.74 on day 20 after treatment initiation (Additional file 1: Figure S1D).

**Fig. 1:**
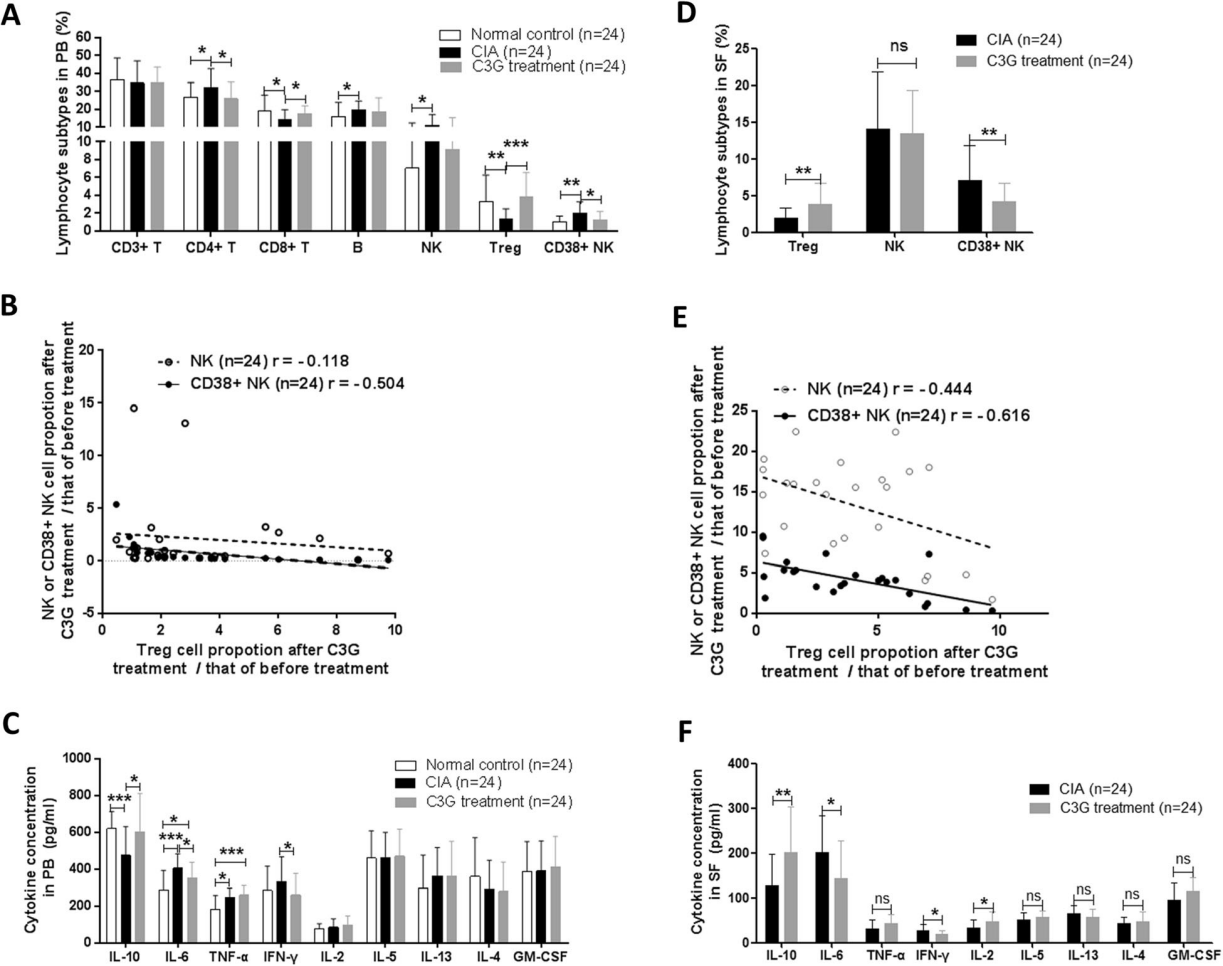
The effect of C3G on lymphocyte subtypes and cytokine secretion in CIA rats using flow cytometry. **a** Proportion of lymphocyte subtypes to total lymphocytes in rat peripheral blood (PB). **b** Correlation of CD38+ natural killer (NK) cell proportion to Treg proportion in rat PB. **c** Cytokine concentration in rat PB. **d** Proportion of lymphocyte subtypes to total lymphocytes in rat synovial fluid (SF). **e** Correlation analysis of CD38+ NK cell proportion to Treg cell proportion in rat SF. **f** Cytokine concentration in rat SF. * indicates *p* < 0.05, **p < 0.01, and ***p < 0.001

Peripheral blood from each group of rats was collected on day 20 after C3G treatment began. Compared with those of the CIA group, the proportions of Treg cells and CD8+ T cells in the C3G treatment group were significantly increased (*p* = 0.0003 and *p* = 0.026, respectively). The proportions of CD4+ T cells and CD38+ NK cells in the C3G treatment group significantly decreased (*p* = 0.033 and *p* = 0.025, respectively). There was no significant difference in the proportions of NK cells, B cells, and T cells between the C3G treatment group and the CIA group (*p* = 0.26, *p* = 0.51, and *p* = 0.87, respectively) (Fig. 1a). There was no correlation (*r* = − 0.118, *p* = 0.5828) between the proportion of NK cells after C3G treatment/the proportion of NK cells before C3G treatment and the proportion of Treg cells, while the proportion of CD38+ NK cells after C3G treatment/the proportion of CD38+ NK cells before C3G treatment was moderately negatively correlated with the proportion of Treg cells (*r* = − 0.504, *p* = 0.012) (Fig. 1b). Compared with that of the CIA group, the concentration of IL-10 in the C3G treatment group increased significantly (*p* = 0.0201), the IL-6 and IFN-γ concentrations decreased significantly (*p* = 0.0322 and *p* = 0.0459, respectively), and the concentrations of TNF-α, IL-2, IL-5, IL-13, IL-4, and GM-CSF were not significantly different between the two groups (*p* = 0.3877, *p* = 0.417, *p* = 0.829, *p* = 0.9798, *p* = 0.1413, and *p* = 0.6529, respectively) (Fig. 1c). The results are also shown in Additional file 7: Table S2.

This study also analyzed the proportions of NK cells, CD38+ NK cells, and Treg cells in the rat synovial lymphocytes. Compared with that of the CIA group, the proportion of Treg cells in synovial lymphocytes from the C3G group significantly increased (*p* = 0.0035), the proportion of NK cells from the C3G group was not significantly different (*p* = 0.7321), and the proportion of CD38+ NK cells from the C3G group decreased significantly (*p* = 0.0093) (Fig. 1d). There was a small negative correlation between the proportion of CD38+ NK cells after C3G treatment/the proportion of CD38+ NK cells before C3G treatment (*r* = − 0.444, *p* = 0.0297), while the proportion of CD38+ NK cells after C3G treatment/the proportion of CD38+ NK cells before C3G treatment was moderately negatively correlated with the proportion of Treg cells (*r* = − 0.616, *p* = 0.0013) (Fig. 1e). Compared with those of the CIA group, the concentrations of IL-10 and IL-2 in the C3G treatment group increased significantly (*p* = 0.0051 and *p* = 0.0353, respectively); the IL-6 and IFN-γ concentrations in the C3G treatment group decreased significantly (*p* = 0.0189 and *p* = 0.0125, respectively), and the concentrations of TNF-α, IL-5, IL-13, IL-4, and GM-CSF in the C3G treatment group were not significantly changed (*p* = 0.0575, *p* = 0.0872, *p* = 0.1627, *p* = 0.3565, and *p* = 0.0694, respectively) (Fig. 1f). The results are also shown in Additional file 8: Table S3.

### Effects of C3G on cultured RASFs

**Fig. 2:**
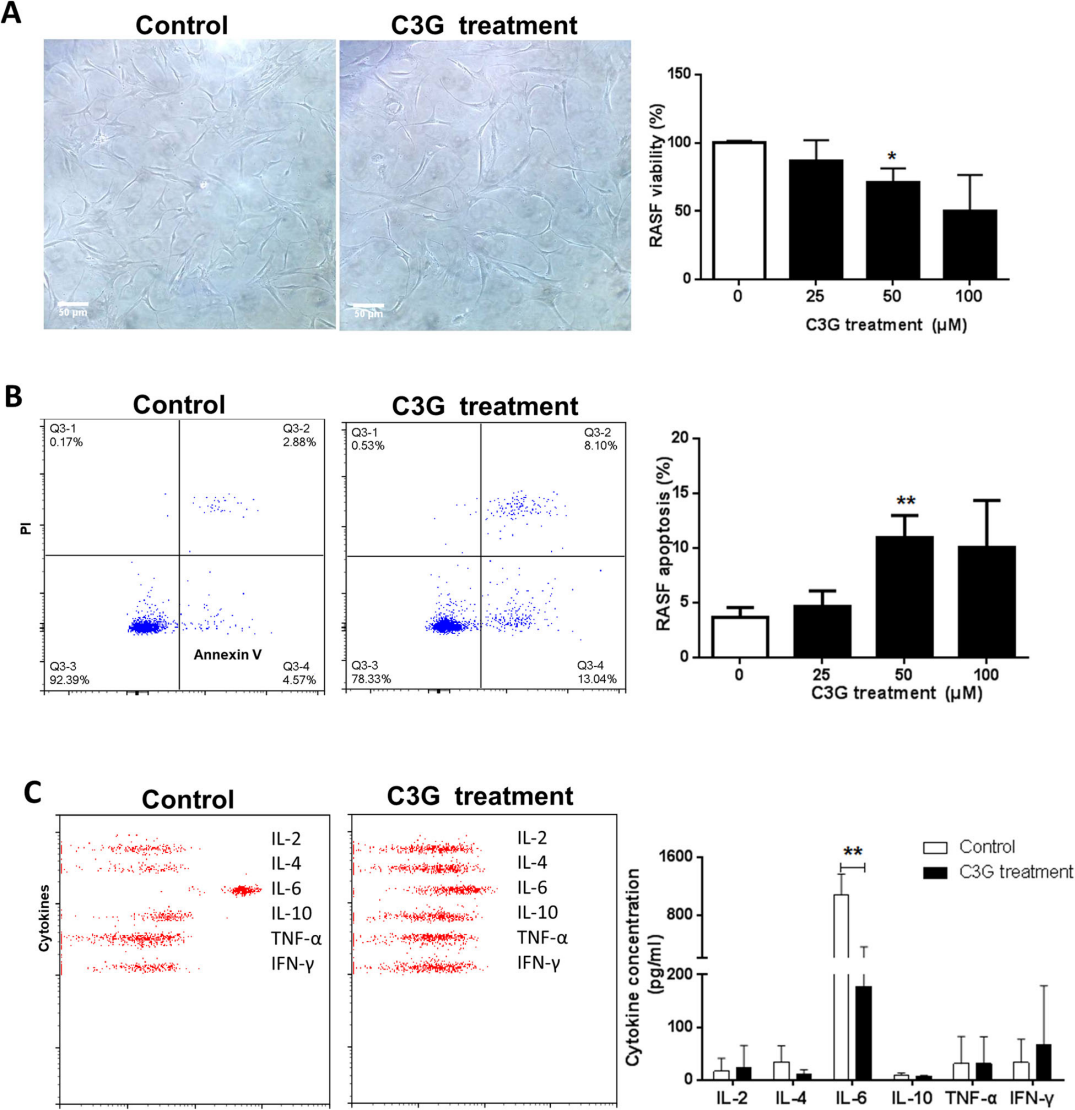
The effect of C3G on rheumatoid arthritis synovial fibroblasts (RASFs). **a** RASF proliferation using CCK8 assay and statistical analysis. **b** RASF apoptosis using flow cytometric Annexin V-FITC/PI assay and statistical analysis. **c** Cytokine production in the culture medium using flow cytometry assay and statistical analysis

This study used C3G to culture RASFs. CCK8 analysis showed that 50 μM C3G inhibited the growth of RASFs to a certain extent (*p* = 0.03) compared with the PBS-treated control group (Fig. 2a). Annexin V/PI apoptosis analysis showed that the apoptosis rate of RASFs increased in the 50 μM C3G group compared with the control group (*p* = 0.07) (Fig. 2b). Flow cytometry analysis of IL-6, IL-2, IL-4, IL-10, TNF-α, and IFN-γ cytokine levels showed that compared with those of the control group (1075 ± 292, 16.94 ± 24.82, 33.88 ± 31.41, 9.493 ± 4.262, 31.39 ± 51.23, 33.37 ± 43.84 pg/mL, respectively), the concentration of IL-6 in the 50 μM C3G group decreased significantly (178 ± 183 pg/mL, *p* = 0.0078) and the concentrations of IL-2, IL-4, IL-10, TNF-α, and IFN-γ were not significantly different (24.77 ± 41.22, *p* = 0.4956; 10.83 ± 8.85, *p* = 0.2189; 6.9 ± 1.97, *p* = 0.4691; 31.19 ± 50.68, *p* = 0.7205; and 66.84 ± 112.4 pg/mL, *p* = 0.4871) (Fig. 2c). The results are also shown in Additional file 9: Table S4.

### Effects of C3G on MNCs

**Fig. 3:**
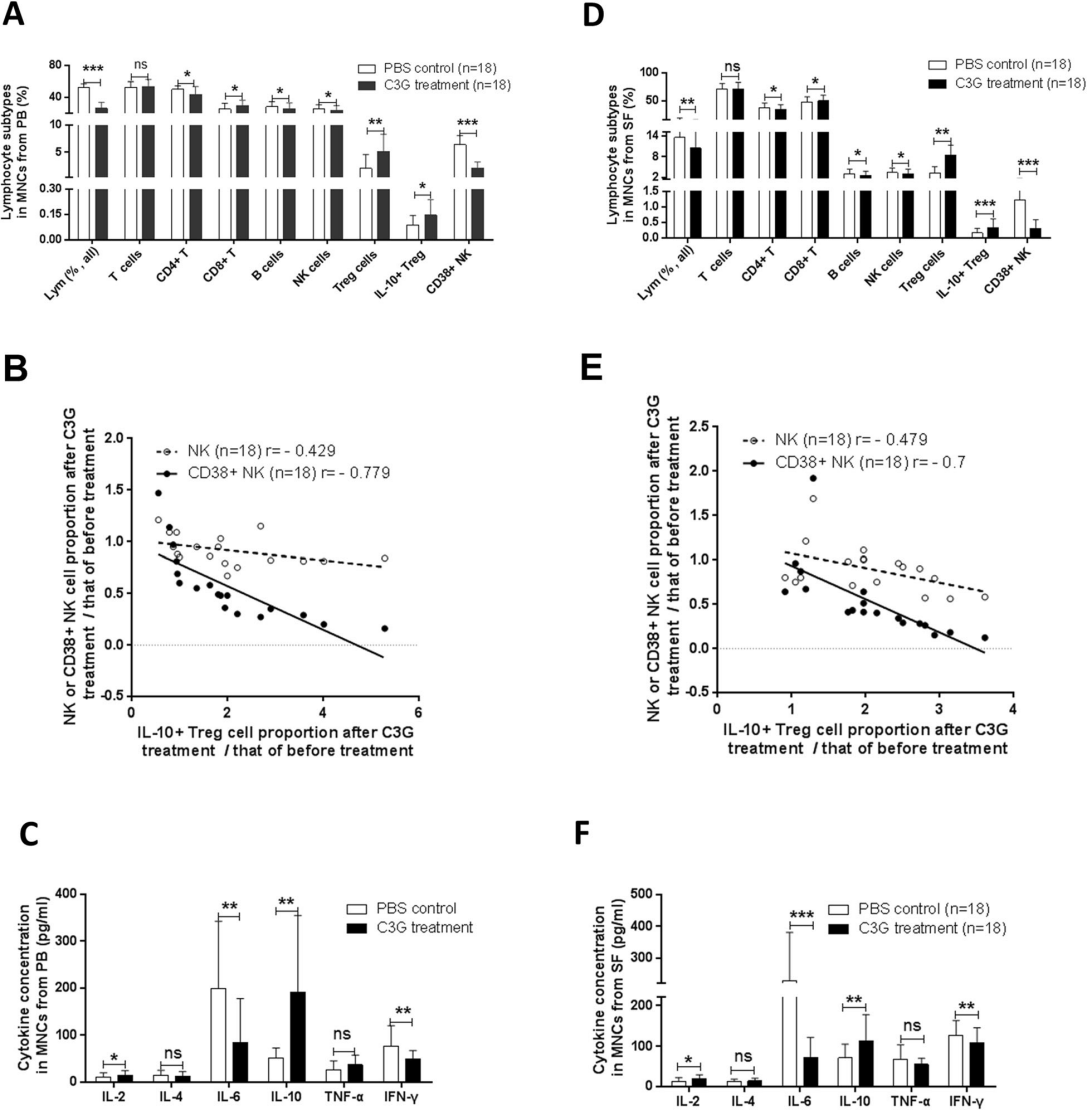
The effect of C3G on lymphocyte subpopulations and cytokine secretion in mononuclear cells (MNCs) using flow cytometry. **a** Proportion of lymphocyte subpopulations within total lymphocytes in MNCs from rheumatoid arthritis (RA) PB. **b** Correlation of NK cells or CD38+ NK cell proportion to interleukin (IL)-10+ Treg proportion in MNCs from PB. **c** Cytokine concentrations in the culture medium of MNCs from PB. **d** Proportion of lymphocyte subpopulations in the total lymphocyte population in MNCs from RA SF. **e** Correlation of NK cells or CD38+ NK cell proportion to IL-10+ Treg proportion in MNCs from SF. **f** Cytokine concentrations in the culture medium of MNCs from SF. * indicates *p* < 0.05, **p < 0.01, and ***p < 0.001

C3G (50 μM) was used to treat MNCs derived from the peripheral blood or synovial fluids of RA patients. In the cultured peripheral blood MNCs compared with those from the PBS-treated control group, the proportions of CD45+ lymphocytes, CD4+ T cells, CD3-CD19+ B cells, and CD3-CD56+ NK cells in the C3G-treated group decreased significantly (*p* < 0.0001, *p* = 0.0152, *p* = 0.0354, *p* = 0.0253); the proportion of T cells in lymphocytes from the C3G-treated group was not significantly different (*p* = 0.4846); the proportions of CD8+ T cells and CD4+ CD25+ Treg cells in lymphocytes from the C3G treatment group increased significantly (*p* = 0.019 and *p* = 0.0031, respectively); the proportion of IL-10+ Treg cells in peripheral blood lymphocytes from the C3G group increased significantly (*p* = 0.0144); and the proportion of CD38+ NK cells decreased significantly (*p* = 0.0001) (Fig. 3a). The results are also shown in Additional file 10: Table S5. Furthermore, the proportion of NK cells after C3G treatment/that before the treatment was not correlated with the proportion of IL-10+ Treg cells (*r* = − 0.429, *p* = 0.0758), and CD38+ NK cells after C3G treatment/those before the treatment were moderately negatively correlated with IL-10+ Treg cells (*r* = − 0.779, *p* = 0.0001) (Fig. 3b). The Treg cell apoptosis in MNCs was measured using flow cytometry. The rate of apoptotic Treg cells was not greatly changed (*p* = 0.31), regardless of whether C3G was present in the culture or not (Additional file 2: Figure S2A).

The concentrations of cytokines in the supernatants of peripheral blood MNC cultures treated with C3G were determined. Compared with those of the PBS control group (198.9 ± 143.5, 77.04 ± 42.82, 10.83 ± 9.007, 51.85 ± 21.23, 15.27 ± 10.25, and 26.42 ± 19.1 pg/mL), the concentrations of IL-6 and IFN-γ in the C3G group decreased significantly (84.39 ± 93.58, *p* = 0.0015; and 49.12 ± 18.59, *p* = 0.0053, respectively), the concentrations of IL-2 and IL-10 in the C3G group increased significantly (14.48 ± 10.68, *p* = 0.0456; 191.8 ± 163.5; *p* = 0.0023), and the concentrations of IL-4 and TNF-α in the C3G group were not significantly different (13.53 ± 9.385, *p* = 0.2298; 37.04 ± 21.11, *p* = 0.1537) (Fig. 3c).

The changes in lymphocyte subsets in RA synovial MNCs after C3G treatment were also measured. Compared with those from the PBS-treated control group, the proportions of CD45+ lymphocytes, CD4+ T cells, CD3-CD19+ B cells, and CD3-CD56+ NK cells in MNCs treated with C3G decreased significantly (*p* = 0.0011, *p* = 0.0208, *p* = 0.0302, and *p* = 0.0345, respectively); the proportion of T cells in the C3G treatment group was not significantly different (*p* = 0.8256), the proportion of CD4+ CD25+ Treg cells in the C3G treatment group increased significantly (*p* = 0.0043); the proportion of IL-10+ Treg cells in synovial MNCs in the C3G-treated group increased significantly (*p* = 0.0006); and the proportion of CD38+ NK cells in synovial MNCs in the C3G-treated group decreased significantly (*p* < 0.0001) (Fig. 3d). The results are shown in Additional file 11: Table S6. Moreover, NK cell proportion after C3G treatment/that before the treatment was weakly correlated with IL-10+ Treg cells (*r* = − 0.479, *p* = 0.0445), and CD38+ NK cell proportion after C3G treatment/that before the treatment was moderately correlated with IL-10+ Treg cells (*r* = − 0.7, *p* = 0.0012) (Fig. 3e).

The concentrations of cytokines in the supernatants of synovial MNC cultures were measured. Compared with the concentration of IL-6, IFN-γ, IL-2, IL-10, IL-4, and TNF- α in the PBS control group (230.1 ± 151.4, 126.5 ± 36.27, 11.95 ± 10.76, 70.91 ± 34.05, 12.11 ± 6.376, and 67.15 ± 36.52 pg/mL, respectively), the concentrations of IL-6 and IFN-γ in the C3G group decreased significantly (71.63 ± 50.07, *p* = 0.0001 and 109 ± 36.07 pg/mL, *p* = 0.0039, respectively), the concentrations of IL-2 and IL-10 in the C3G group increased significantly (19.4 ± 9.974, *p* = 0.0344 and 113.7 ± 63.661, *p* = 0.0078, respectively), and the concentrations of IL-4 and TNF-α in the C3G group were not significantly different (14.24 ± 6.303, *p* = 0.1155 and 54.97 ± 15.19, *p* = 0.1222, respectively) (Fig. 3f).

We sorted CD4+ CD25+ Treg cells using flow cytometry to a purity of 99.84%. C3G was used to stimulate the purified Treg cells. Compared to PBS (11.01 ± 4.881%), treatment with 25 μM (12.2 ± 2.757%), 50 μM (11.72 ± 5.388%), and 100 μM (12.24 ± 4.736%) C3G had no significant effects on the proportion of IL-10+ Treg cells (*p* = 0.6565, *p* = 0.6457, and *p* = 0.0809, respectively) (Additional file 3: Figure S3).

### Effects of C3G-treated CD38+ NK cells on MNCs

**Fig. 4:**
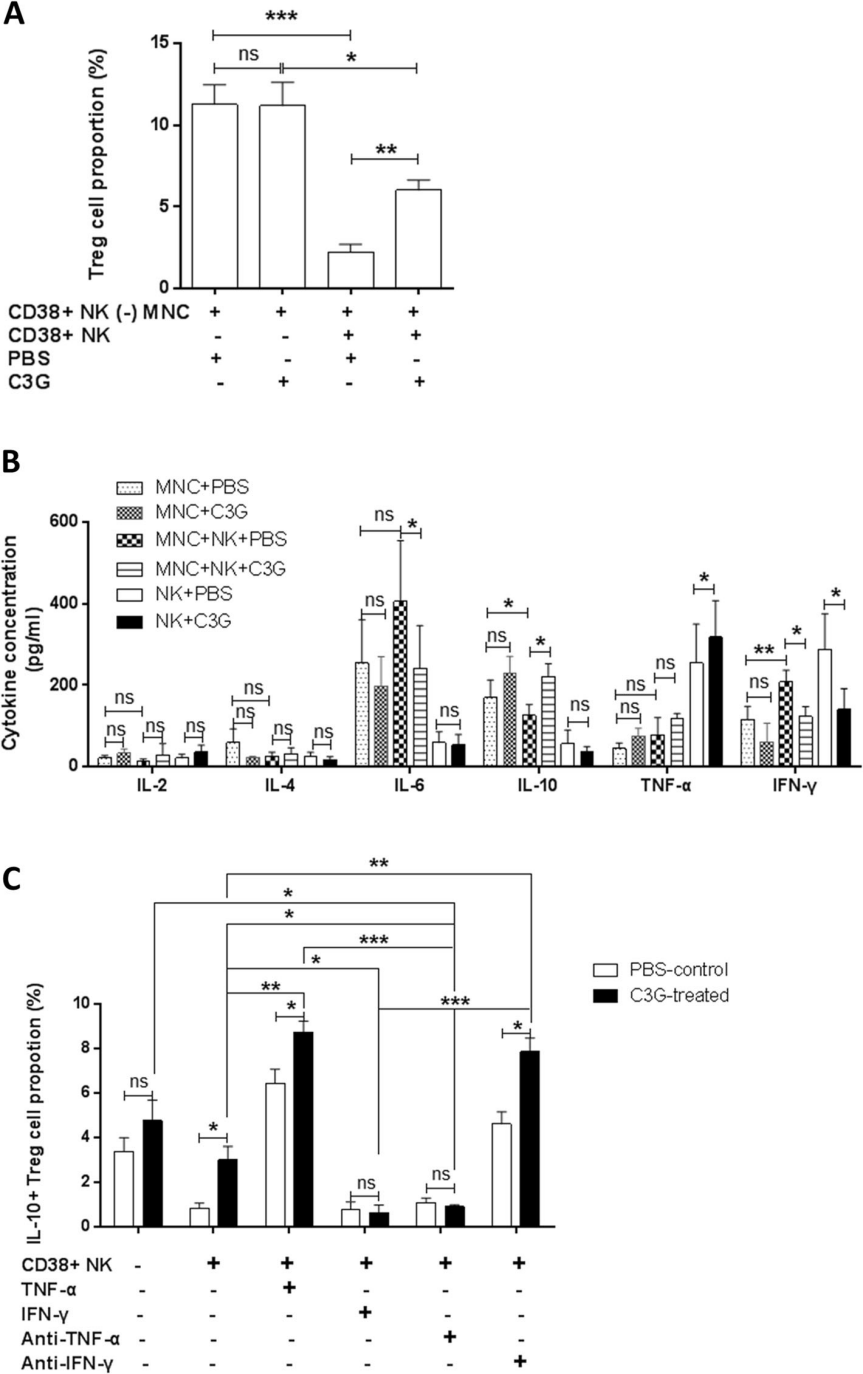
The effect of CD38+ NK and C3G on MNCs. **a** Treg cell proportion in MNCs depleted of CD38+ NK cells (CD38+ NK cells (−) MNC). **b** Cytokine production in medium of cocultured CD38+ NK and MNCs. **c** IL-10+ Treg proportion in MNCs depleted of CD38+ NK cells. The MNCs were cocultured with CD38+ NK cells following treatment with tumor necrosis factor (TNF)-α, interferon (IFN)-γ, anti-TNF-α, anti-IFN-γ, and C3G. * indicates *p* < 0.05

We sorted CD38+ NK cells and MNCs depleted of CD38+ NK cells from peripheral blood of RA patients. The purity of CD38+ CD3− CD56+ NK cells was 99.23%. The CD38+ NK cells were treated with C3G and were then cocultured with MNCs depleted of CD38+ NK cells in separated chambers. Compared with that of the control group in which the MNCs were cultured alone (11.27 ± 2.66%), the proportion of Treg cells in MNCs treated directly with C3G was not significantly different (11.18 ± 3.22%, *p* = 0.96), and the proportion of Treg cells in the MNCs cocultured with CD38+ NK cells decreased significantly (2.22 ± 1.08%, *p* = 0.0001). Compared with MNCs that were cocultured with CD38+ NK cells (2.22 ± 1.08%), the proportion of Treg cells in the MNCs cocultured with C3G-pretreated CD38+ NK cells increased significantly (6 ± 1.42%, *p* = 0.0015) (Fig. 4a).

We measured apoptosis of Treg cells in MNCs following coculture with CD38+ NK cells in a transwell apparatus. We found that the rate of apoptotic Treg cells was not greatly changed (*p* = 0.15), regardless of whether CD38+ NK cells were pretreated with C3G or not (Additional file 2: Figure S2B). We also cocultured CD38+ NK cells and MNCs in the same well and then measured CD38+ NK cell-mediated cytotoxicity against Treg cells. Susceptibility of Treg cells to lysis mediated by CD38+ NK cells was slightly reduced (*p* = 0.03) when CD38+ NK cells were pretreated with C3G (Additional file 4: Figure S4).

Compared with those of the control group in which untreated MNCs were cultured alone, the concentrations of IL-2, IL-4, IL-6, IL-10, TNF-α, and IFN-γ in the MNCs directly treated with C3G were not significantly different (*p* = 0.2235, *p* = 0.1909, *p* = 0.6155, *p* = 0.1811, *p* = 0.1243, and *p* = 0.2749, respectively). Compared with MNCs cocultured with untreated CD38+ NK cells (405.8 ± 150, 207.5 ± 28.9, and 126.1 ± 26.75 pg/mL, respectively), the concentrations of IL-6 and IFN-γ in MNCs cocultured with C3G-pretreated CD38+ NK cells decreased significantly (241.4 ± 104.6, *p* = 0.0351 and 123.3 ± 24.25, *p* = 0.035, respectively), the concentration of IL-10 increased significantly (221 ± 31.25, *p* = 0.0393), and the concentrations of IL-2, IL-4, and TNF-α were not significantly different (*p* = 0.4134, *p* = 0.5512, and *p* = 0.3088, respectively). Compared with those of the control group in which untreated CD38+ NK cells were cultured alone, the concentrations of IL-2, IL-4, IL-6, and IL-10 in the culture medium of C3G-treated CD38+ NK cells were not significantly different (*p* = 0.2931, *p* = 0.1396, *p* = 0.8760, and *p* = 0.5299, respectively). Compared with that of the control group in which untreated CD38+ NK cells were cultured alone (254.5 ± 95.41, 288.1 ± 87.19 pg/mL), the concentration of TNF-α in the culture medium of C3G-pretreated CD38+ NK cells increased significantly (317.6 ± 90.071, *p* = 0.0231) and the concentration of IFN-γ decreased significantly (140.9 ± 49.91, *p* = 0.0367). Compared with the control group in which MNCs were cultured alone (169.2 ± 42.64, 115.7 ± 31.96 pg/mL), the concentration of IL-10 in MNCs cocultured with CD38+ NK cells decreased significantly (126.1 ± 26.75, *p* = 0.0429), the concentration of IFN-γ increased significantly (207.5 ± 28.91, *p* = 0.0035), and the concentrations of IL-2, IL-4, IL-6, and TNF-α were not significantly different (*p* = 0.3721, *p* = 0.2579, *p* = 0.3803, and *p* = 0.3475, respectively) (Fig. 4b).

To determine whether IFN-γ and TNF-α are contributing to CD38+ NK cells’ influence on Treg differentiation of MNCs, CD38+ NK cells were pretreated with 50 μM C3G and cocultured with MNCs that were depleted of CD38+ NK cells. TNF-α (10 ng/mL), IFN-γ (5 ng/mL), anti-TNF-α antibody, or anti-IFN-γ antibody was added to the culture medium. In the absence of CD38+ NK cells, C3G treatment did not significantly change the proportion of IL-10+ Treg cells in MNCs (*p* = 0.6192). Compared with that of the PBS treatment control (0.85 ± 0.38), the proportion of IL-10+ Treg cells in MNCs cocultured with C3G-pretreated CD38+ NK cells significantly increased (3.01 ± 1.03, *p* = 0.0265). Compared with MNCs cocultured with CD38+ NK cells pretreated with C3G (3.01 ± 1.03), the proportion of IL-10+ Treg cells in MNCs cocultured with CD38+ NK cells pretreated with C3G significantly increased (8.72 ± 0.88, *p* = 0.0018) in the presence of TNF-α in the culture medium, whereas the proportion of IL-10+ Treg cells in MNCs cocultured with CD38+ NK cells pretreated with C3G decreased significantly (0.61 ± 0.63, *p* = 0.0261) in the presence of IFN-γ in the culture medium. Compared with MNCs cultured with C3G alone (4.76 ± 1.60), MNCs cocultured with C3G-pretreated CD38+ NK cells and TNF-α (8.72 ± 0.88), and MNCs cocultured with C3G-pretreated CD38+ NK cells alone (3.01 ± 1.03), the proportion of IL-10+ Treg cells in MNCs cocultured with C3G-pretreated CD38+ NK cells and anti-TNF-α antibody significantly decreased (0.92 ± 0.13, *p* = 0.048, *p* = 0.0001, and *p* = 0.024, respectively). Compared with those of MNCs cocultured with C3G-pretreated CD38+ NK cells alone (3.01 ± 1.03) or MNCs cocultured with C3G-pretreated CD38+ NK cells and IFN-γ (0.61 ± 0.63), the proportion of IL-10+ Treg cells in MNCs cocultured with C3G-pretreated CD38+ NK cells and anti-IFN-γ antibody significantly increased (7.86 ± 1.05, *p* = 0.0046 and *p* = 0.0005, respectively) (Fig. 4c). The results are also shown in Additional file 12: Table S7.

### Effects of Sirt6 expression on CD38+ NK cells

**Fig. 5:**
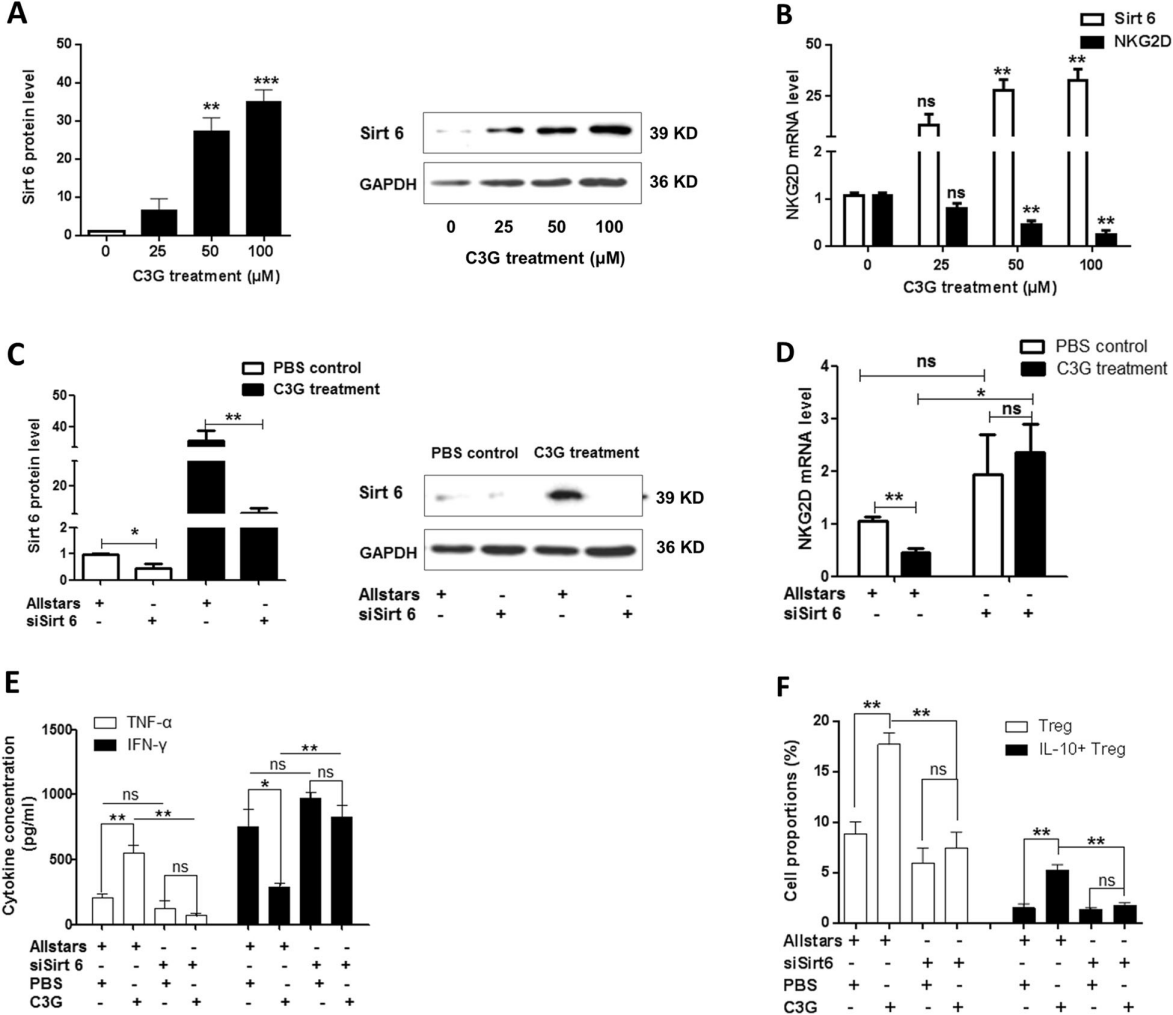
Sirtuin6 (Sirt6) expression and its effect on CD38+ NK cells following C3G treatment. **a** Sirt6 expression level in CD38+ NK cells following C3G treatment using real-time PCR and Western blotting. **b** NKG2D mRNA level in CD38+ NK cells following C3G treatment using real-time PCR. **c** Sirt6 expression level in CD38+ NK cells following Sirt6 siRNA (siSirt 6) transfection with Allstars siRNA as a control using real-time PCR and Western blotting. **d** NKG2D mRNA level in CD38+ NK cells following Sirt6 siRNA transfection using real-time PCR. **e** IFN-γ and TNF-α concentration in the culture medium of CD38+ NK cells following treatment with Sirt6 siRNA and C3G. **f** Treg and IL-10+ Treg cell proportions in CD38+ NK-depleted MNCs. The MNCs were cocultured with CD38+ NK cells treated with Sirt6 siRNA and C3G. * indicates *p* < 0.05, **p < 0.01, and ***p < 0.001

We treated CD38+ NK cells with C3G to investigate its molecular mechanism. Compared with PBS treatment (1.06 ± 0.1217), 50 μM C3G (27.33 ± 6.110) and 100 μM C3G (35.00 ± 5.568) treatment significantly increased the expression of Sirt6 mRNA (*p* = 0.0017 and *p* = 0.0005, respectively) in CD38+ NK cells. Western blotting also showed that C3G treatment increased the expression levels of Sirt6 protein (Fig. 5a). Compared with the PBS control, 50 μM C3G and 100 μM C3G treatment significantly decreased the expression of NKG2D mRNA (*p* = 0.0054 and *p* = 0.0019, respectively) (Fig. 5b).

Sirt6 siRNA was transfected into CD38+ NK cells. The expression of Sirt6, as determined by real-time PCR and western blotting, significantly decreased after the interference (Fig. 5c). Compared with that of the control group transfected with Allstars control siRNA alone, the expression of NKG2D mRNA in CD38+ NK cells treated with C3G and Allstars siRNA significantly decreased (*p* = 0.0054). Compared with that of the control group treated only with Sirt6 siRNA, the expression of NKG2D mRNA in CD38+ NK cells treated with C3G and Sirt6 siRNA was not significantly different (*p* = 0.6697) (Fig. 5d).

Compared with the concentrations of TNF-α and IFN-γ in the control group treated with Allstars siRNA alone (205 ± 51.97 and 756.7 ± 228.1, respectively), C3G treatment significantly increased the concentration of TNF-α (556 ± 94.98, *p* = 0.0049) and decreased the concentration of IFN-γ (289.3 ± 48.52, *p* = 0.0255) in the supernatant of CD38+ NK cells. Compared with those of the control group treated with Sirt6 siRNA alone, the levels of TNF-α and IFN-γ in CD38+ NK cells treated with both Sirt6 siRNA and C3G were not significantly changed (*p* = 0.3857 and *p* = 0.2284, respectively). Compared with the TNF-α and IFN-γ concentrations in the control group treated with C3G alone (556 ± 94.98 and 289.3 ± 48.52, respectively), the level of TNF-α in CD38+ NK cells treated with both Sirt6 siRNA and C3G decreased significantly (75.67 ± 21.5, *p* = 0.001), and the level of IFN-γ in C3G-treated cells increased significantly (828.7 ± 161.1, *p* = 0.0051) (Fig. 5e).

Compared to that in MNCs cocultured with CD38+ NK cells that were treated with both C3G and Allstars siRNA, the proportion of Treg cells and IL-10+ Treg cells in MNCs cocultured with CD38+ NK cells that were treated with both Sirt6 siRNA and C3G was significantly decreased (*p* = 0.0056 and *p* = 0.0067, respectively). When CD38+ NK cells were transfected with Sirt6 siRNA, the proportion of Treg cells and IL-10+ Treg cells in MNCs did not significantly change, regardless of C3G treatment (Fig. 5f).

### Effects of a Sirt6 inhibitor on CIA in rats

**Fig. 6:**
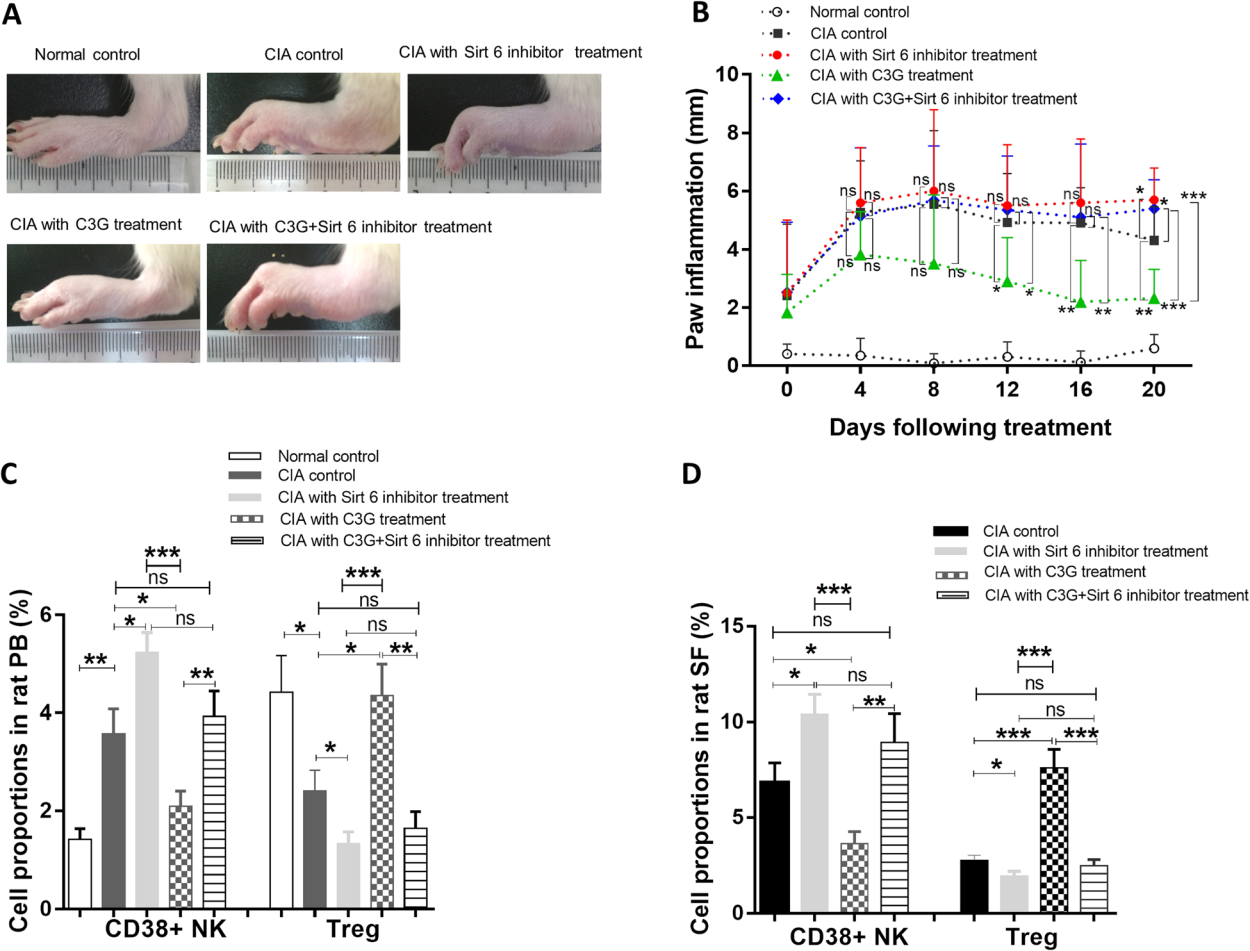
The effect of Sirt6 inhibitor on rat CIA with C3G treatment. **a** Joint inflammation of CIA rats treated with C3G and Sirt6 inhibitor. **b** Inflammation curve analysis based on paw swelling. **c** CD38+ NK cell proportion and Treg cell proportion within the total lymphocytes in rat PB. **d** CD38+ NK cell proportion and Treg cell proportion within the total lymphocytes in rat SF. * indicates *p* < 0.05, **p < 0.01, and ***p < 0.001

C3G and OSS_128167, a Sirt6 inhibitor, were injected into the CIA rats. Compared with the CIA group, toe swelling in CIA rats treated with C3G was significantly alleviated (*p* = 0.0013), but the toes of CIA rats were still swollen or a little worse after treatment with both C3G and OSS_128167 (*p* = 0.049) or only OSS_128167 (*p* = 0.019). Compared with the C3G alone-treated group, the toe swelling of CIA rats treated with both C3G and Sirt6 inhibitor or the inhibitor alone increased significantly (*p* < 0.0001, *p* < 0.0001, respectively) (Fig. 6a, b). Additionally, compared with that of the CIA group, the proportion of CD38+ NK cells in CIA rats treated with C3G decreased significantly (*p* = 0.022) in peripheral blood, the proportion was not changed in CIA rats treated with both C3G and Sirt6 inhibitor (*p* = 0.638), and the proportion was even elevated in CIA rats treated with the Sirt6 inhibitor alone (*p* = 0.021). On the other hand, compared with that of the C3G group, the proportion of Treg cells in the group treated with both C3G and Sirt6 inhibitor or the inhibitor alone decreased significantly (*p* = 0.0019 and *p* = 0.0005, respectively) (Fig. 6c). The results are also shown in Additional file 13: Table S8.

Compared with the CIA controls, the proportion of CD38+ NK cells in the synovial fluid decreased significantly following C3G treatment (*p* = 0.019), but the proportion in CIA rats treated with both C3G and Sirt6 inhibitor was not significantly changed (*p* = 0.264). Meanwhile, the proportion of Treg cells increased significantly (*p* = 0.0002) in CIA rats after C3G treatment, and the proportion was not significantly changed after treatment of either C3G and Sirt6 inhibitor treatment or the inhibitor alone (*p* = 0.5414, *p* = 0.042). Compared with C3G treatment group, the proportion of CD38+ NK cells increased significantly in CIA rats treated with both C3G and Sirt6 inhibitor or inhibitor alone (*p* = 0.005 and *p* = 0.0001, respectively). Meanwhile, the proportion of Treg cells decreased significantly in CIA rats treated with both C3G and Sirt6 inhibitor or inhibitor alone (*p* = 0.0001 and *p* = 0.0004) (Fig. 6d). The results are also shown in Additional file 14: Table S9.

## Discussion

This study explored whether C3G has therapeutic effects on RA using CIA rats, in vitro cultured RASFs, and MNCs as models. The inflammation curves and histochemical analyses showed that inflammation in CIA rats was significantly relieved after C3G injection. Moreover, C3G injection reduced the concentrations of the inflammatory cytokine IL-6 and IFN-γ and increased the levels of the anti-inflammatory cytokine IL-10 in the peripheral blood and synovial fluid of CIA rats. Following C3G treatment, RASFs showed decreased proliferation, increased apoptosis, and reduced IL-6 secretion. We also found that C3G increased the proportion of Treg cells in peripheral blood and synovial fluid of CIA rats. We used C3G to culture MNCs that were derived from the peripheral blood and synovial fluid of patients with RA. C3G elevated the proportions of Treg cells and IL-10+ Treg cells in MNCs from RA patients. Treg cells participate in negative immune regulation by secreting cytokines such as IL-10 and TGF-β, inhibiting various immune cells, and playing immunoregulatory functions in autoimmune and allergic immune diseases [39–41]. IL-10 secreted from Treg cells exerts anti-inflammatory effects by inhibiting Th cell transformation, B cell activation, NK cell activity, and cytokine secretion [42–44]. IL-10 plays important roles in many autoimmune diseases such as RA [45]. It has been reported that the ability of Treg cells to produce IL-10 is impaired in patients with RA [46]. In RA patients, the number of Treg cells is negatively correlated with DAS28—the RA disease activity score—and Treg cells inhibit excessive immune activation through secreting IL-10 [46, 47]. Therefore, the possibility of treating RA with Treg cells and IL-10 has been proposed [47, 48]. The above in vivo and in vitro results demonstrate that C3G has therapeutic effects on CIA and RA and suggest that C3G may exert its therapeutic effect by increasing the proportion of Treg cells and their IL-10 secretion and decreasing RASF proliferation as well as proinflammatory cytokine production.

We examined changes in the proportions of CD38+ NK cells in the peripheral blood and synovial fluid of each group of rats. The number of CD38+ NK cells in CIA rats increased, and the proportion of CD38+ NK cells decreased after C3G injection. Moreover, the ratio of the proportion of CD38+ NK cells after C3G treatment to the proportion of CD38+ NK cells before C3G treatment was negatively correlated with the ratio of the proportion of Treg cells after C3G treatment to the proportion of Treg cells before C3G treatment. We also found that C3G reduced the proportions of total lymphocytes and CD4+ T, NK, B, CD8+ T, and CD38+ NK cells and increased the proportions of Treg and IL-10+ Treg cells in MNCs. Moreover, the ratio of the proportion of CD38+ NK cells after C3G treatment to the proportion of CD38+ NK cells before C3G treatment was negatively correlated with the ratio of the proportion of IL-10+ Treg cells after C3G treatment to the proportion of IL-10+ Treg cells before C3G treatment. C3G simultaneously increased IL-2 and IL-10 secretion and decreased IL-6 and IFN-γ secretion in the MNC culture medium. It has been reported that NK cells inhibit the proliferation, differentiation, and functions of Treg cells [37, 49–51]. Our results suggest that C3G may achieve therapeutic effects against RA and CIA by reducing the proportion of CD38+ NK cells to stimulate proportional changes in IL-10+ Treg cells and IL-10 secretion.

To further investigate the cellular mechanism of C3G on MNCs and Treg differentiation, we treated Treg cells with C3G. We found that C3G did not directly change Treg cell proportions or their IL-10 secretion. Additionally, C3G treatment did not directly change the proportion of Treg cells in MNCs depleted of CD38+ NK cells, and there were no changes in the concentrations of IL-2, IL-4, IL-6, IL-10, TNF-α, and IFN-γ in the culture medium. However, the proportion of Treg cells increased in MNCs cocultured with C3G-treated CD38+ NK cells, the concentrations of IL-6 and IFN-γ decreased, the concentration of IL-10 increased, and the proportion of Treg cells in MNCs cocultured with CD38+ NK was reduced. These results further demonstrate that C3G does not directly act on MNCs to mediate Treg cell differentiation. C3G increases the proportion of Treg cells in MNCs by decreasing the proportion of CD38+ NK cells, thereby increasing IL-10 secretion, decreasing IL-6 and IFN-γ levels, and achieving therapeutic effects on RA. Additionally, we examined apoptosis of Treg cells in MNCs following coculture with CD38+ NK cells in a transwell apparatus. We found that the rate of apoptotic Treg cells did not change greatly whether CD38+ NK cells were pretreated with C3G or not. We also examined apoptosis of Treg cells in MNCs following coculture with CD38+ NK cells in a transwell. We found that the rate of apoptotic Treg cells was not greatly changed. The results demonstrated that C3G did not affect Treg cell apoptosis in MNCs. Thus, the increase in Treg cell numbers in MNCs cocultured with C3G-pretreated CD38+ NK cells may be mainly due to increased Treg differentiation rather than decreased cell death sensitivity.

Sirt6 is an NAD+-dependent deacetylase. In vitro studies have shown that Sirt6 adenovirus reduces the inflammatory responses and tissue destruction of CIA by blocking the NF-κB pathway, downregulating local and systemic levels of proinflammatory factors, and inhibiting macrophages to induce osteoclast differentiation [30, 31]. It has been found that CD38 inhibition is an effective way to promote sirtuin activation [52, 53]. We treated CD38+ NK cells with C3G and found that the mRNA and protein level of Sirt6 increased after treatment, while the mRNA NKG2D level significantly decreased. NKG2D acts as a powerful activating and costimulatory receptor on immune effector cells including NK and T cells [54]. Disruptions within the NKG2D signaling pathway may trigger an exacerbated immune response and promote autoimmune reactions [55–57]. In our study, when MNCs were cocultured with CD38+ NK cells with pretreatment of both Sirt6 siRNA and C3G, the proportions of Treg cells and IL-10+ Treg cells were significantly decreased compared with those in MNCs cocultured with CD38+ NK cells treated with C3G alone. Compared with the control group which was treated with Sirt6 siRNA alone, the NKG2D mRNA expression level in CD38+ NK cells treated with both C3G and Sirt6 siRNA did not change significantly. These results suggest that C3G increases Sirt6 expression and subsequently decreases NKG2D expression in CD38+ NK cells. In *Sirt6* (+/−) *ApoE* (−/−) mice, Sirt6 downregulation increases expression of NKG2D ligands, which leads to increased cytokine expression. Blocking the NKG2D ligand almost completely blocks this effect [58], which is consistent with our observations.

OSS_128167 (SIRT6-IN-1, C19H14N2O6) is a cell-permeable and Sirt6-selective inhibitor [22, 59, 60]. We injected CIA rats with C3G in combination with OSS_128167. The toes of the CIA rats remained swollen after treatment with both C3G and Sirt6 inhibitor, while the proportion of Treg cells in the CIA rats remained low. The proportion of CD38+ NK cells in CIA rats decreased after C3G treatment or after treatment with both C3G and OSS_128167, and there was no significant difference in the proportions of CD38+ NK cells between the two groups. The animal experiment further supports that C3G attenuates the progression of CIA in rats via regulating Sirt6 expression in CD38+ NK cells. Sirt6 inhibitor did not affect the number of CD38+ NK cells, but it blocked the therapeutic effects of C3G on CIA by reducing Sirt6 activity, which decreases the proportion of Treg cells.

This study found that the concentration of TNF-α increased and the concentration of IFN-γ decreased in the medium of CD38+ NK cells treated with C3G. When MNCs were cocultured with C3G-pretreated CD38+ NK cells, the proportion of IL-10+ Treg cells increased significantly in MNCs in the presence of TNF-α or C3G and anti-IFN-γ antibody, while the proportion decreased when MNCs were cocultured with C3G-pretreated CD38+ NK cells in the presence of IFN-γ or C3G and anti-TNF-α antibody. Furthermore, there was no significant change in the secretion of TNF-α and IFN-γ in the C3G-treated CD38+ NK cells after transfection with Sirt6 siRNA, indicating that CD38+ NK cells mediate TNF-α and IFN-γ secretion through regulating Sirt6 expression. These results suggest that C3G stimulates the differentiation of IL-10+ Treg cells in MNCs by enhancing Sirt6 expression to promote TNF-α secretion and inhibit IFN-γ secretion in CD38+ NK cells.

Studies have shown that NK cells exacerbate the inflammatory responses of RA by secreting IFN-γ, and CD38 can promote the IFN-γ secretion by NK cells [61–63]. IFN-γ inhibits the differentiation of Treg cells, and TNF-α promotes the activation of Treg cells [64, 65]. It has been reported that Sirt6 promotes TNF-α secretion, and Sirt6 directly upregulates TNF-α secretion via defatty-acylation [66]. However, a recent study found that NKG2D signaling also regulates TNF-α release by NK cells. NKG2D ligand interaction in NK cells increases the activity of the metalloprotease TNF-α-converting enzyme [67]. Another study reported that IFN-γ, TNF-α, perforin, and granzyme B levels were partially blocked by NKG2D mAb [55]. Considering our study and others, we hypothesize that C3G stimulates Sirt6 expression to directly elevate TNF-α expression. The increased Sirt6 expression by C3G may simultaneously downregulate NKG2D to mediate TNF-α and IFN-γ. Overall, C3G upregulates TNF-α and downregulates IFN-γ production in CD38+ NK cells through increasing Sirt6 expression.

We detected decreased expression of NKG2D in CD38+ NK cells following C3G treatment. NKG2D is a major recognition receptor for the detection and elimination of transformed and infected cells as its ligands are induced during cellular stress, either as a result of infection or genomic stress such as in cancer. In NK cells, NKG2D serves as an activating receptor and is itself able to trigger cytotoxicity. NKG2D+ CD4+ T cells efficiently kill NKG2D ligand (NKG2DL)+ Treg cells [56]. We cocultured CD38+ NK cells and MNCs in two separate chambers in a transwell apparatus. Although some Treg cells express NKG2D ligands [56], it is unlikely that CD38+ NK cells directly killed Treg cells in the separate transwell compartments by intercellular contact. We also examined cytotoxicity of CD38+ NK cells against Treg cells with coculture. We found that susceptibility of Treg cells to CD38+ NK cell-mediated lysis decreased slightly when CD38+ NK cells were pretreated with C3G. This result indicated that CD38+ NK cell has mild cytotoxicity to Treg cells through cell-to-cell contact, and C3G can block this killing ability in the cell. Although the current data cannot exclude the possibility that the reduced killing of Tregs by CD38+ NK cells is involved in vivo, CD38+ NK cells play an essential role on Treg cell differentiation through non-intercellular contact by mediating TNF-α and IFN-γ secretion. Additionally, we observed a decreased proportion of CD38+ NK cells in the CIA model and cultured MNCS following C3G treatment. Thus, we consider that there are three possibilities by which the Treg proportion is increased by CD38+ NK cells following C3G treatment in vivo: (1) decreased CD38+ NK cell proportion after C3G treatment, although we do not yet completely understand the mechanism, (2) increased TNF-α secretion and decreased IFN-γ secretion in CD38+ NK cells following C3G treatment to induce Treg cell differentiation by non-intercellular contact, and (3) decreased Treg cell cytotoxicity mediated by intercellular contact with CD38+ NK cells following C3G treatment.

## Conclusions

This study demonstrates that C3G has therapeutic effects on CIA and RA. The study also demonstrates that CD38+ NK cell-mediated inhibition of Treg cell differentiation in MNCs is a potential cause of the immune imbalance in RA and CIA. Our results suggest a mechanism in which C3G decreases CD38+ NK and RASF cell proportions and simultaneously increases Sirt6 expression in CD38+ NK cells, which inhibits the expression of NKG2D and results in increased TNF-α secretion and decreased IFN-γ secretion in CD38+ NK cells. Due to both the reduced proportion of CD38+ NK cells and increased Sirt6 expression, MNCs are stimulated by C3G to increase the proportion of IL-10+ Treg cells and IL-10 secretion, leading to the improvements in CIA and RA symptoms (Additional file 5: Figure S5). The above findings suggest the possibility of C3G becoming a therapeutic drug for RA.

## Supplementary information

**Additional file 1: Figure S1.** The effect of cyanidin-3-O-glucoside (C3G) on rat collagen-induced arthritis (CIA). (A) Joint inflammation of CIA rats treated with C3G. (B) Histochemical observation of rat joint tissue. (C) Inflammation curve analysis based on paw swelling. (D) Disease scores were quantified based on histologic evidence. * indicates *p* < 0.05, ** *p* < 0.01, and *** *p* < 0.001.

**Additional file 2: Figure S2.** The effect of C3G and CD38+ NK cells on apoptosis of Treg cells from RA peripheral MNCs. (A) Apoptosis of Treg cells in MNCs with C3G treatment. (B) Apoptosis of Treg cells in MNCs following coculture with CD38+ NK cells in a transwell apparatus.

**Additional file 3: Figure S3.** The effect of C3G on Treg cell proportion in RA peripheral MNCs. IL-10+ Treg cell proportion in Treg cells following C3G treatment.

**Additional file 4: Figure S4.** Cytotoxicity assay of CD38+ NK cells against Treg cells from RA peripheral MNCs in the presence of C3G.

**Additional file 5: Figure S5.** Schematic explaining the pathogenic pathway of CD38+ NK cells and therapeutic mechanism of C3G in RA. C3G decreases the CD38+ NK cell proportion. C3G also increases Sirt6 expression in CD38+ NK cells, which inhibits NKG2D expression, and simultaneously stimulates TNF-α secretion and reduces IFN-γ secretion. As a result, the proportion of IL-10+ Treg cells and IL-10 secretion are elevated in MNCs, thereby exerting a therapeutic effect on RA. The dotted line indicates a suggestion based on studies of others.

**Additional file 6: Table S1.** Detailed clinical information of patients with RA (DOC 132 kb)

**Additional file 7: Table S2.** Lymphocyte subset proportion (%) and cytokine levels (pg/mL) in peripheral blood of CIA rats.

**Additional file 8: Table S3.** Lymphocyte subset proportion (%) and cytokine levels (pg/mL) in CIA synovial fluid.

**Additional file 9: Table S4.** Cytokine levels (pg/mL) in synovial fibroblast-like cells.

**Additional file 10: Table S5.** The proportions (%) of lymphocyte subsets in MNCs from peripheral blood.

**Additional file 11: Table S6.** The proportions of CD45+ lymphocyte subsets in MNCs from synovial fluid.

**Additional file 12: Table S7.** The proportions of IL-10+ Treg cells in MNCs depleted of CD38+ NK cells.

**Additional file 13: Table S8.** Lymphocyte subset proportion (%) in peripheral blood of CIA rats.

**Additional file 14: Table S9.** Lymphocyte subset proportion (%) in CIA synovial fluid.

## Data Availability

All relevant data and materials are included in this published article.

## Abbreviations

APC: Allophycocyanin
C3G: Cyanidin-3-O-glucoside
cADPR: Cyclic ADP-ribose
cADPRH: Cyclic ADP-ribose hydrolase
CIA: Collagen-induced arthritis
DAS28: Disease Activity Score
GM-CSF: Granulocyte-macrophage colony-stimulating factor
HE: Hematoxylin-eosin
IFN-γ: Interferon-γ
IL: Interleukin
MNC: Mononuclear cell
NAD+: Nicotinamide adenine dinucleotide
NC: Normal control
NK: Natural killer
NKG2D: Natural killer group 2, member D
PBS: Phosphate-buffered saline
PCR: Polymerase chain reaction
PE: Phycoerythrin
PerCP: Peridinin chlorophyll protein
PMNC: Peripheral blood mononuclear cell
RA: Rheumatoid arthritis
RASF: Rheumatoid arthritis synovial fibroblast
Sirt6: Sirtuin 6
SMNC: Synovial fluid mononuclear cell
TGF-β: Transforming growth factor-β
TNF-α: Tumor necrosis factor-α
Treg: T regulatory
