# Supplementary figures and images for: Potential therapeutic effects of cyanidin-3-O-glucoside on rheumatoid arthritis by relieving inhibition of CD38+ NK cells on Treg cell differentiation
Source: Arthritis Res Ther. 2019 Oct 28;21:220. doi: 10.1186/s13075-019-2001-0 (PMC6819496; doi:10.1186/s13075-019-2001-0)

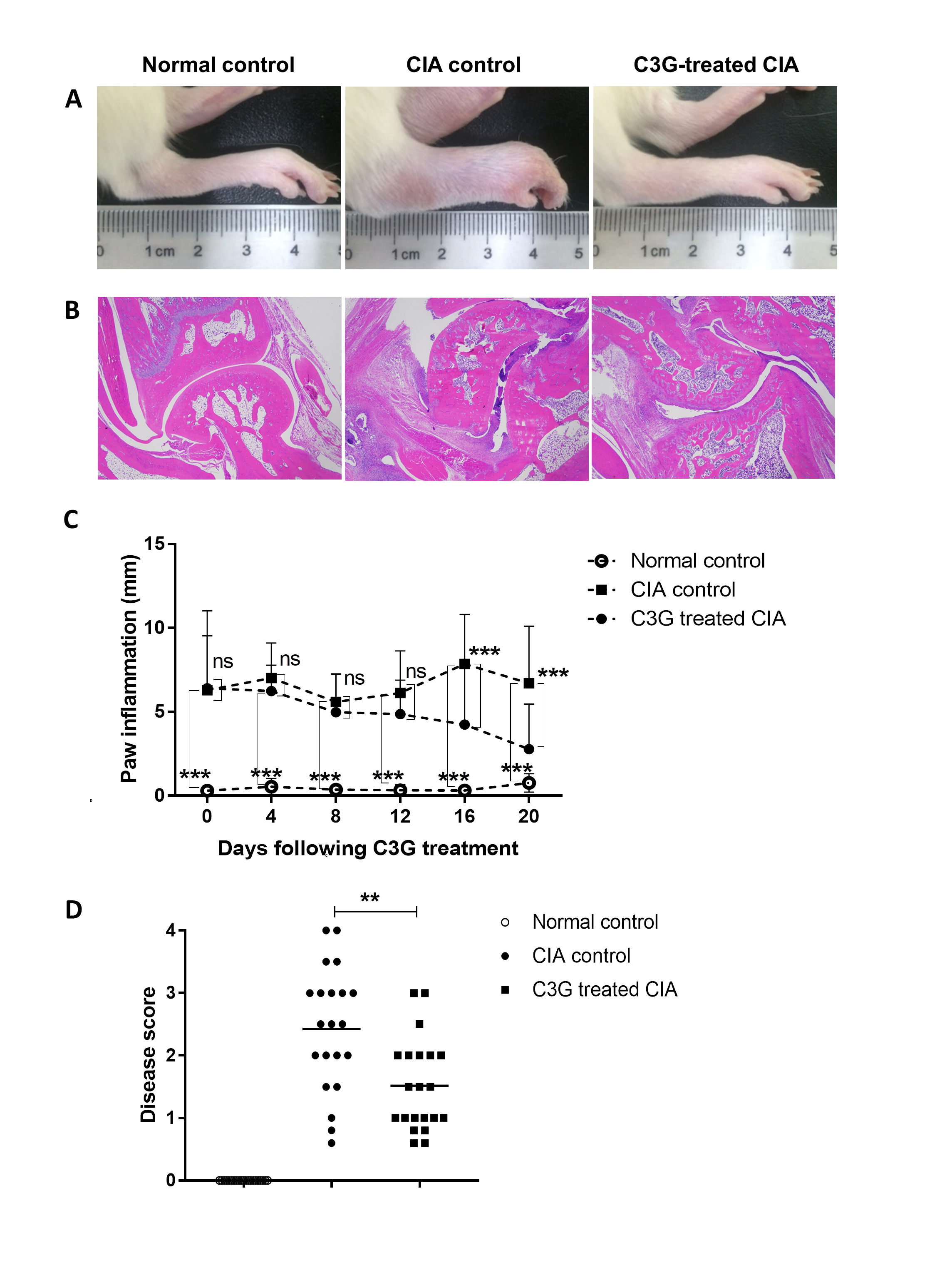

Supplement: Supplementary file 1 — Additional file 1: Figure S1. The effect of cyanidin-3-O-glucoside (C3G) on rat collagen-induced arthritis (CIA). (A) Joint inflammation of CIA rats treated with C3G. (B) Histochemical observation of rat joint tissue. (C) Inflammation curve analysis based on paw swelling. (D) Disease scores were quantified based on histologic evidence. * indicates p < 0.05, ** p < 0.01, and *** p < 0.001. [file 13075_2019_2001_MOESM1_ESM.tif]

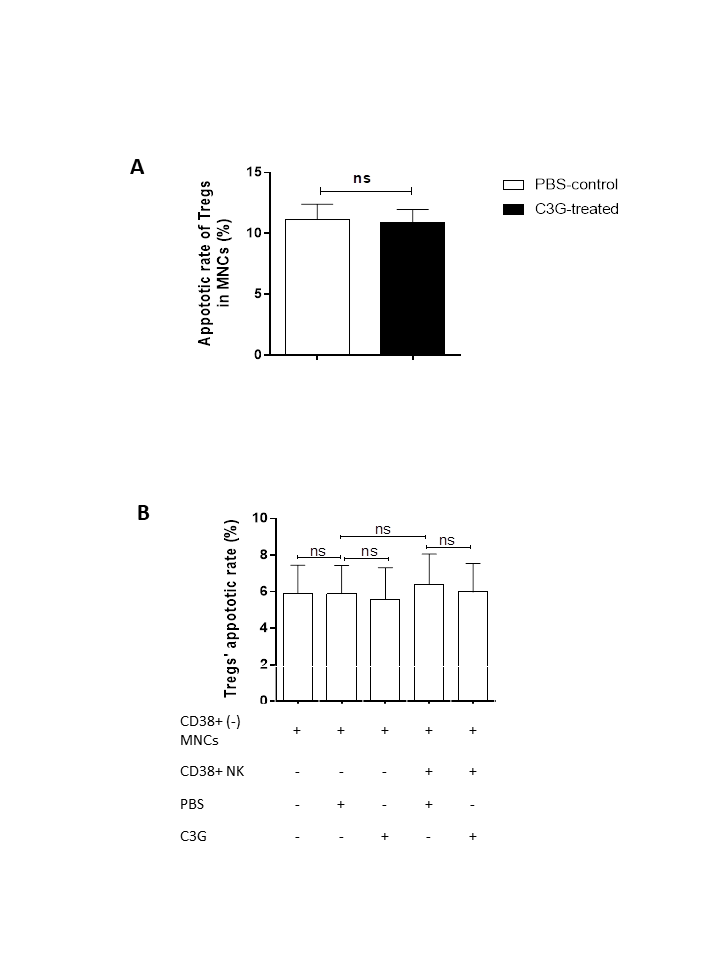

Supplement: Supplementary file 2 — Additional file 2: Figure S2. The effect of C3G and CD38+ NK cells on apoptosis of Treg cells from RA peripheral MNCs. (A) Apoptosis of Treg cells in MNCs with C3G treatment. (B) Apoptosis of Treg cells in MNCs following coculture with CD38+ NK cells in a transwell apparatus. [file 13075_2019_2001_MOESM2_ESM.tif]

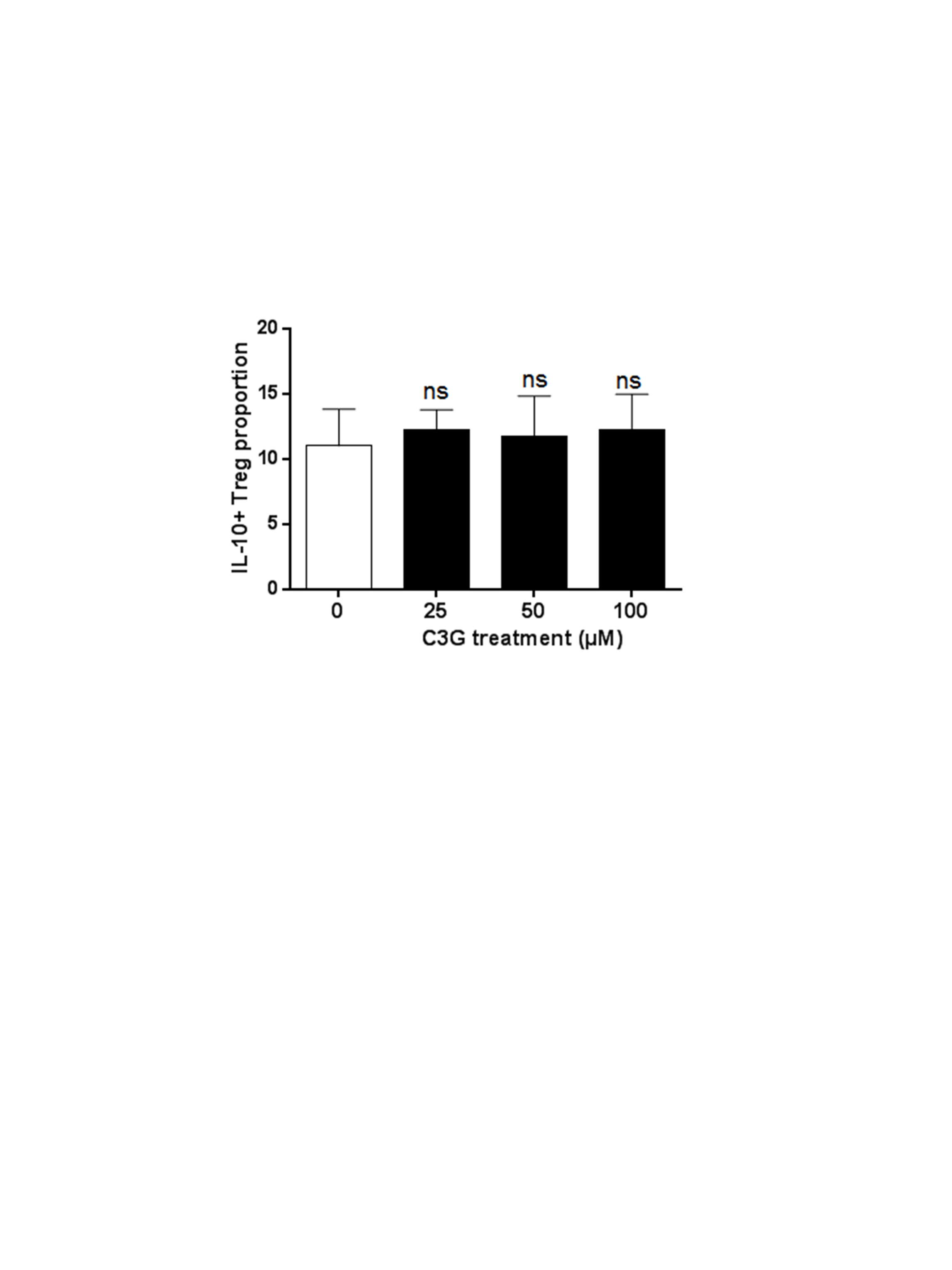

Supplement: Supplementary file 3 — Additional file 3: Figure S3. The effect of C3G on Treg cell proportion in RA peripheral MNCs. IL-10+ Treg cell proportion in Treg cells following C3G treatment. [file 13075_2019_2001_MOESM3_ESM.tif]

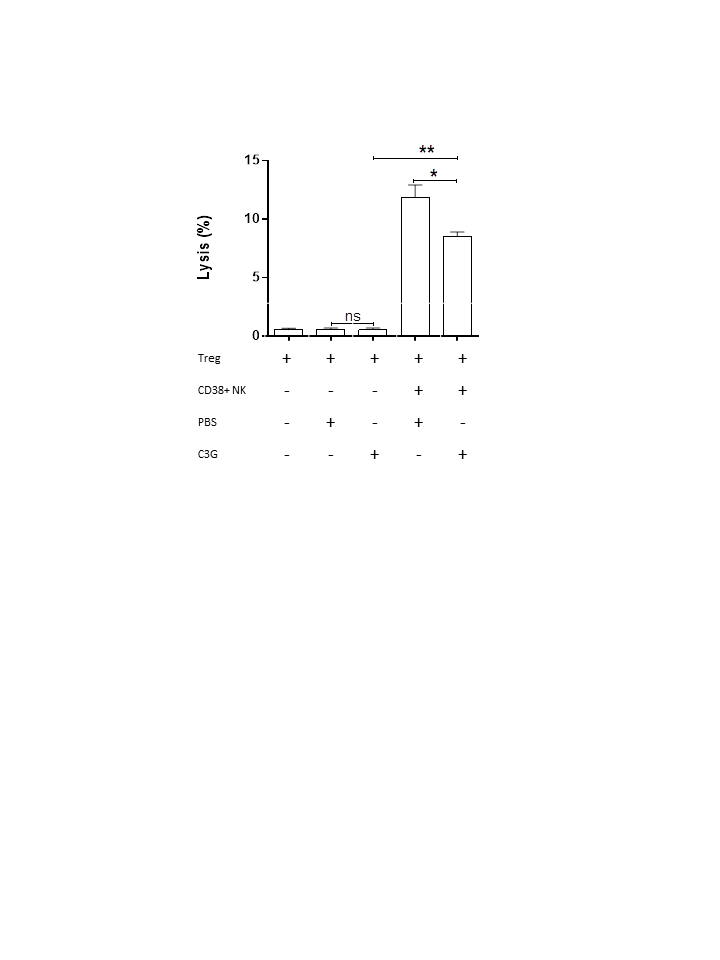

Supplement: Supplementary file 4 — Additional file 4: Figure S4. Cytotoxicity assay of CD38+ NK cells against Treg cells from RA peripheral MNCs in the presence of C3G. [file 13075_2019_2001_MOESM4_ESM.tif]

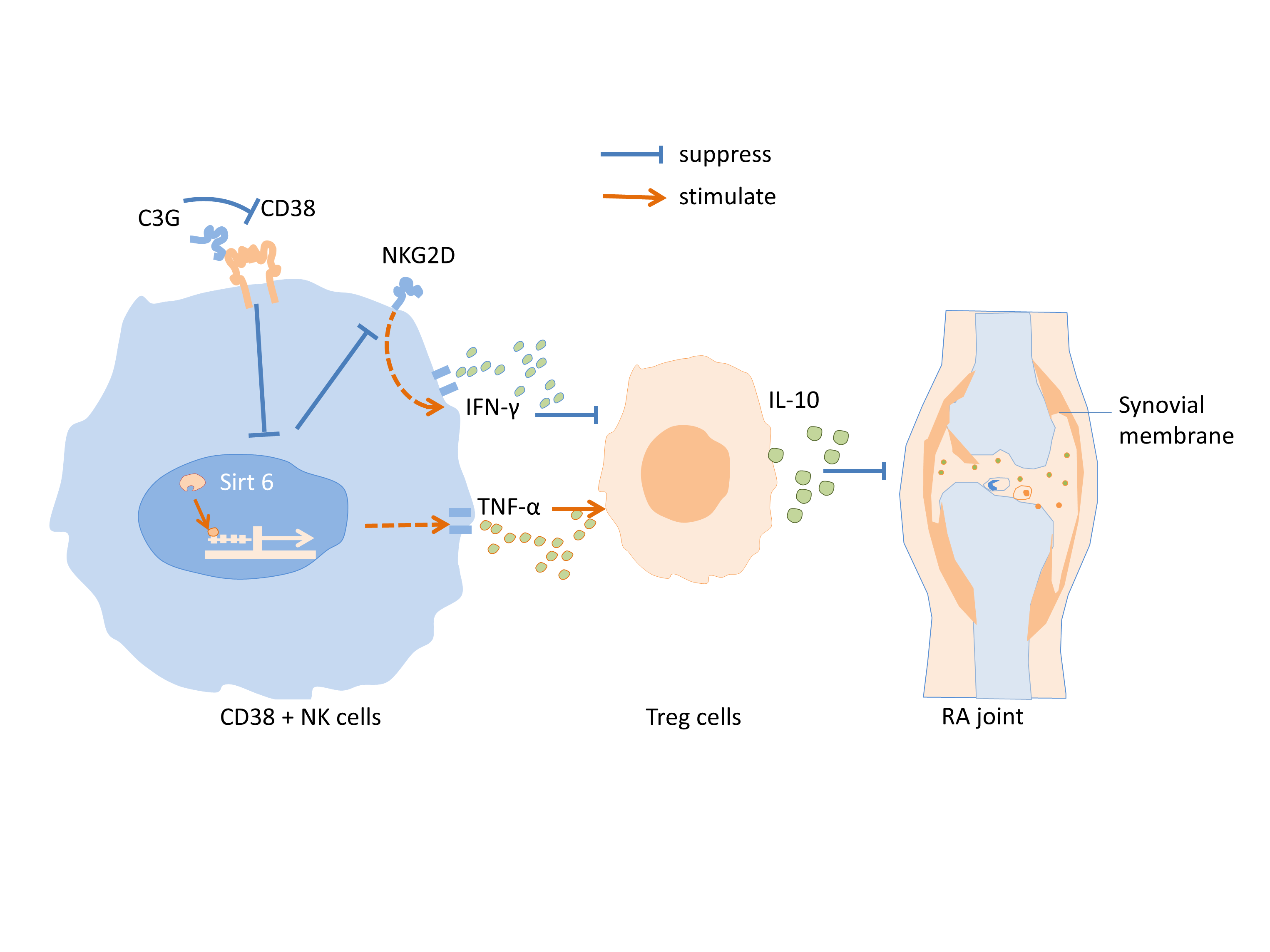

Supplement: Supplementary file 5 — Additional file 5: Figure S5. Schematic explaining the pathogenic pathway of CD38+ NK cells and therapeutic mechanism of C3G in RA. C3G decreases the CD38+ NK cell proportion. C3G also increases Sirt6 expression in CD38+ NK cells, which inhibits NKG2D expression, and simultaneously stimulates TNF-α secretion and reduces IFN-γ secretion. As a result, the proportion of IL-10+ Treg cells and IL-10 secretion are elevated in MNCs, thereby exerting a therapeutic effect on RA. The dotted line indicates a suggestion based on studies of others. [file 13075_2019_2001_MOESM5_ESM.tif]
