# Supplementary material for: Potential therapeutic effects of cyanidin-3-O-glucoside on rheumatoid arthritis by relieving inhibition of CD38+ NK cells on Treg cell differentiation
Source: Arthritis Res Ther. 2019 Oct 28;21:220. doi: 10.1186/s13075-019-2001-0 (PMC6819496; doi:10.1186/s13075-019-2001-0)
Supplement: Supplementary file 6 — Additional file 6: Table S1. Detailed clinical information of patients with RA (DOC 132 kb) [file 13075_2019_2001_MOESM6_ESM.doc]

**Table S1. Detailed clinical information of patients with RA**

| **NO.** | **Gender** | **Age** | **Duration of disease** | **RF (IU)** | **DAS28** | **CRP (mg/L)** | **Anti-CCP (U)** | **Treatment agents** | **Sample type** |
| --- | --- | --- | --- | --- | --- | --- | --- | --- | --- |
| 1 | female | 42 | 5 | 64 | 4.7 | 29 | 65 | DMARDs | 1 |
| 2 | female | 81 | 2 | 162 | 5.4 | 84 | 101 | DMARDs | 1 |
| 3 | female | 51 | 3 | 134 | 4.2 | 61 | 86 | DMARDs, TCM | 1 |
| 4 | female | 59 | 0.6 | 30 | 3.2 | 4.3 | 54 | DMARDs | 1 |
| 5 | female | 21 | 9 | 67 | 5.1 | 78 | 91 | DMARDs, TCM | 1 |
| 6 | female | 48 | 1 | 86 | 4.5 | 66 | 75 | DMARDs | 1 |
| 7 | female | 58 | 3 | 56 | --- | 3 | 46 | DMARDs | 1 |
| 8 | female | 62 | 1 | 78 | 3.6 | 5.6 | 46 | DMARDs | 1 |
| 9 | female | 65 | 7 | 361 | 5.5 | 43 | 73 | DMARDs | 1 |
| 10 | female | 67 | 10 | 329 | 5.1 | 102 | 130 | DMARDs, NSAIDs | 1 |
| 11 | female | 58 | 8 | 152 | 4.3 | 11 | 78 | DMARDs | 1 |
| 12 | female | 54 | 10 | 153 | 6.4 | 121 | 132 | DMARDs | 1 |
| 13 | female | 45 | 1 | 78 | 4.8 | 34 | 64 | DMARDs | 1 |
| 14 | female | 25 | 1 | 54 | 5 | 36 | 85 | DMARDs, TCM | 1, 2 |
| 15 | male | 78 | 2 | 53 | 3.8 | 0.4 | 39 | DMARDs | 1, 2 |
| 16 | female | 74 | 20 | 759 | 7.1 | 129 | 157 | DMARDs, NSAIDs | 1, 2 |
| 17 | female | 68 | 20 | 724 | 5.8 | 167 | 201 | DMARDs, TCM | 1, 2 |
| 18 | female | 61 | 10 | 59 | 3.9 | 46 | 63 | DMARDs, NSAIDs | 1, 2 |
| 19 | female | 38 | 8 | 121 | 4.6 | 42 | 56 | DMARDs | 1, 2 |
| 20 | male | 63 | 5 | 77 | 4.4 | 38 | 81 | DMARDs, NSAIDs | 1, 2 |
| 21 | male | 51 | 2 | 87 | 4.2 | 43 | 77 | DMARDs | 1, 2 |
| 22 | female | 54 | 30 | 753 | 6.4 | 45 | 82 | DMARDs | 1, 2 |
| 23 | female | 68 | 1 | 631 | 5.7 | 79 | 106 | DMARDs | 1, 2 |
| 24 | female | 54 | 9 | 213 | 4.2 | 57 | 111 | DMARDs, TCM | 1, 2 |
| 25 | female | 38 | 15 | 436 | 5.4 | 97 | 67 | DMARDs | 1, 2 |
| 26 | female | 54 | 7 | 365 | 6.1 | 108 | 137 | DMARDs, NSAIDs | 1, 2 |
| 27 | male | 62 | 8 | 243 | 6.3 | 56 | 87 | DMARDs, NSAIDs | 1, 2 |
| 28 | female | 66 | 6 | 654 | 7.7 | 122 | 355 | DMARDs, NSAIDs | 1, 2, 3 |
| 29 | female | 72 | 20 | 476 | --- | 132 | 476 | DMARDs, NSAIDs | 1, 2, 3 |
| 30 | female | 54 | 7 | 198 | 7.1 | 108 | 1200 | DMARDs, NSAIDs | 1, 2, 3 |
| 31 | female | 74 | 12 | 327 | 6.4 | 92 | 853 | DMARDs, NSAIDs | 2, 3 |
| 32 | female | 77 | 15 | 565 | 6.2 | 73 | 568 | DMARDs, TCM | 2, 3 |
| 33 | male | 54 | 5 | 634 | 6.9 | 116 | 464 | DMARDs, NSAIDs | 2, 3 |
| 34 | female | 23 | 3 | 579 | 6.6 | 98 | 550 | DMARDs, NSAIDs | 3 |

RA: rheumatoid arthritis; CRP: C-reactive protein; anti-CCP: anticyclic citrullinated peptide; DAS28: Disease Activity Score 28; DMARDs: disease-modifying anti-rheumatic drugs; NSAIDs: nonsteroidal anti-inflammatory drugs; TCM: traditional Chinese medicine; sample type: “1” indicates peripheral blood; “2” indicates synovial fluid; “3” indicates synovial membrane tissues.
