# Supplementary material for: Potential therapeutic effects of cyanidin-3-O-glucoside on rheumatoid arthritis by relieving inhibition of CD38+ NK cells on Treg cell differentiation
Source: Arthritis Res Ther. 2019 Oct 28;21:220. doi: 10.1186/s13075-019-2001-0 (PMC6819496; doi:10.1186/s13075-019-2001-0)
Supplement: Supplementary file 7 — Additional file 7: Table S2. Lymphocyte subset proportion (%) and cytokine levels (pg/mL) in peripheral blood of CIA rats. [file 13075_2019_2001_MOESM7_ESM.doc]

**Table S2. Lymphocyte subset proportion (%) and cytokine levels (pg/mL) in peripheral blood of CIA rats**

|  | **NC group** | **CIA group** | **C3G-treated group** | **CIA vs C3G** | **NC vs CIA** |
| --- | --- | --- | --- | --- | --- |
| **CD38+ NK cells** | 1.04±0.65 | 2.01±1.27 | 1.27±0.93 | p=0.025 | p=0.0016 |
| **Treg** | 3.29±2.99 | 1.37±2.99 | 3.78±2.80 | p=0.0003 | p=0.005 |
| **CD4+ T** | 26.72±8.32 | 32.29±10.44 | 26.02±9.33 | p=0.033 | p=0.046 |
| **CD8+T** | 19.07±9.01 | 14.42±5.56 | 17.72±4.27 | p=0.026 | p=0.037 |
| **NK cells** | 7.04±5.41 | 11.15±6.01 | 9.12±6.35 | p=0.26 | p=0.016 |
| **B cells** | 15.99±7.96 | 19.97±4.79 | 18.73±7.80 | p=0.51 | p=0.042 |
| **T cells** | 36.39±12.26 | 34.6±12.43 | 35.12±8.53 | p=0.87 | p=0.62 |
| **IL-2** | 79.1±26.6 | 86.3±46.0 | 97.9±49.9 | p=0.417 | p=0.519 |
| **IL-4** | 360.8±210.4 | 292.8±156.4 | 280.1±159.6 | p=0.1413 | p=0.210 |
| **IL-5** | 460.7±147.9 | 462.6±137.6 | 471.5±146.4 | p=0.829 | p=0.9629 |
| **IL-6** | 288.4±105.2 | 405.8±79.0 | 353.9±83.8 | p=0.0322 | p<0.0001 |
| **IL-10** | 620.9±93.0 | 475.3±155.8 | 603.1±208.2 | p=0.0201 | p=0.0003 |
| **IL-13** | 296.5±180.5 | 364.2±154.0 | 363.0±189.1 | p=0.9798 | p=0.168 |
| **TNF-α** | 183.7±75.4 | 247.4±50.14 | 260.4±52.5 | p=0.3877 | p=0.0012 |
| **GM-CSF** | 389.4±161.1 | 391.5±162.6 | 413.0±165.6 | p=0.6529 | p=0.9632 |
| **IFN-γ** | 285.8±130.4 | 331.5±136.8 | 260.7±116.8 | p=0.0459 | p=0.214 |
