# Supplementary material for: Potential therapeutic effects of cyanidin-3-O-glucoside on rheumatoid arthritis by relieving inhibition of CD38+ NK cells on Treg cell differentiation
Source: Arthritis Res Ther. 2019 Oct 28;21:220. doi: 10.1186/s13075-019-2001-0 (PMC6819496; doi:10.1186/s13075-019-2001-0)
Supplement: Supplementary file 8 — Additional file 8: Table S3. Lymphocyte subset proportion (%) and cytokine levels (pg/mL) in CIA synovial fluid. [file 13075_2019_2001_MOESM8_ESM.docx]

**Table S3. Lymphocyte subset proportion (%) and cytokine levels (pg/mL) in CIA synovial fluid**

|  | **CIA group** | **C3G-treated group** | **P value** |
| --- | --- | --- | --- |
| **Treg** | 2.018±1.287 | 3.873±2.809 | 0.0035 |
| **NK** | 14.14±7.752 | 13.45±5.858 | 0.7321 |
| **CD38+NK** | 7.077±4.701 | 4.240±2.509 | 0.0093 |
| **IL-2** | 33.93±18.12 | 46.82±22.79 | 0.0353 |
| **IL-4** | 43.±15.15 | 48.02±21.63 | 0.3565 |
| **IL-5** | 66.±17.97 | 56.73±18.75 | 0.0872 |
| **IL-6** | 201.5±81.80 | 143.8±82.61 | 0.0189 |
| **IL-10** | 127.6±69.80 | 201.8±102 | 0.0051 |
| **IL-13** | 51.73±14.9 | 57.49±13.17 | 0.1627 |
| **TNF-α** | 31.26±20.86 | 42.73±19.95 | 0.0575 |
| **GM-CSF** | 95.16±38.78 | 114.6±31.49 | 0.0694 |
| **IFN-γ** | 28.31±14.00 | 20.12±6.468 | 0.0125 |
