# Supplementary material for: Potential therapeutic effects of cyanidin-3-O-glucoside on rheumatoid arthritis by relieving inhibition of CD38+ NK cells on Treg cell differentiation
Source: Arthritis Res Ther. 2019 Oct 28;21:220. doi: 10.1186/s13075-019-2001-0 (PMC6819496; doi:10.1186/s13075-019-2001-0)
Supplement: Supplementary file 9 — Additional file 9: Table S4. Cytokine levels (pg/mL) in synovial fibroblast-like cells. [file 13075_2019_2001_MOESM9_ESM.docx]

**Table S4. Cytokine levels (pg/mL) in synovial fibroblast-like cells**

|  | **Control group** | **C3G group** | **P value** |
| --- | --- | --- | --- |
| **IL-2** | 16.94±24.82 | 24.77±41.22 | 0.4956 |
| **IL-4** | 33.88±31.41 | 10.83±8.85 | 0.2189 |
| **IL-6** | 1075±292 | 178±183 | 0.0078 |
| **IL-10** | 9.493±4.262 | 6.9±1.97 | 0.4691 |
| **TNF-α** | 31.39±51.23 | 31.19±50.68 | 0.7205 |
| **IFN-γ** | 33.37±43.84 | 66.84±112.4 | 0.4871 |
