# Supplementary material for: Potential therapeutic effects of cyanidin-3-O-glucoside on rheumatoid arthritis by relieving inhibition of CD38+ NK cells on Treg cell differentiation
Source: Arthritis Res Ther. 2019 Oct 28;21:220. doi: 10.1186/s13075-019-2001-0 (PMC6819496; doi:10.1186/s13075-019-2001-0)
Supplement: Supplementary file 10 — Additional file 10: Table S5. The proportions (%) of lymphocyte subsets in MNCs from peripheral blood. [file 13075_2019_2001_MOESM10_ESM.docx]

**Table S5. The proportions (%) of lymphocyte subsets in MNCs from peripheral blood**

|  | **PBS control** | **C3G treatment** | **P value** |
| --- | --- | --- | --- |
| **CD45+ lymphocytes** | 52.54±4.52 | 26.00±7.73 | p<0.0001 |
| **CD4+ T cells** | 50.01±4.56 | 43.61±10.13 | 0.0152 |
| **CD3- CD19+ B cells** | 28.24±6.35 | 25.8±7.22 | 0.0354 |
| **CD3- CD56+ NK cells** | 25.68±5.07 | 23.5±6.14 | 0.0253 |
| **T cells** | 52.5±7.74 | 53.73±8.49 | 0.4846 |
| **CD8+ T cells** | 25.16±7.34 | 29.36±7.01 | 0.019 |
| **CD4+ CD25+ Treg cells** | 2.01±2.57 | 5.08±3.23 | 0.0031 |
| **IL-10+ Treg cells** | 0.087±0.056 | 0.14±0.09 | 0.0144 |
| **CD38+ NK cells** | 6.38±1.69 | 2.10±1.12 | 0.0001 |
