# Supplementary material for: Potential therapeutic effects of cyanidin-3-O-glucoside on rheumatoid arthritis by relieving inhibition of CD38+ NK cells on Treg cell differentiation
Source: Arthritis Res Ther. 2019 Oct 28;21:220. doi: 10.1186/s13075-019-2001-0 (PMC6819496; doi:10.1186/s13075-019-2001-0)
Supplement: Supplementary file 11 — Additional file 11: Table S6. The proportions of CD45+ lymphocyte subsets in MNCs from synovial fluid. [file 13075_2019_2001_MOESM11_ESM.docx]

**Table S6. The proportions of CD45+ lymphocyte subsets in MNCs from synovial fluid**

|  | **PBS control** | **C3G treatment** | **P value** |
| --- | --- | --- | --- |
| **CD45+ lymphocytes** | 13.45±5.234 | 10.39±4.703 | 0.0011 |
| **CD4+ T cells** | 36.97±8.427 | 34.49±8.409 | 0.0208 |
| **CD3- CD19+ B cells** | 3±1.339 | 2.458±1.299 | 0.0302 |
| **CD3- CD56+ NK cells** | 3.359±1.283 | 2.907±1.391% | 0.0345 |
| **T cells** | 71.3±9.87 | 71.51±11.66 | 0.8256 |
| **CD4+ CD25+ Treg cells** | 3.208±1.845 | 8.468±2.774 | 0.0043 |
| **IL-10+ Treg cells** | 0.1611±0.1460 | 0.3200±0.2944 | 0.0006 |
| **CD38+ NK cells** | 1.214±0.473 | 0.302±0.279 | <0.0001 |
