# Supplementary material for: Potential therapeutic effects of cyanidin-3-O-glucoside on rheumatoid arthritis by relieving inhibition of CD38+ NK cells on Treg cell differentiation
Source: Arthritis Res Ther. 2019 Oct 28;21:220. doi: 10.1186/s13075-019-2001-0 (PMC6819496; doi:10.1186/s13075-019-2001-0)
Supplement: Supplementary file 12 — Additional file 12: Table S7. The proportions of IL-10+ Treg cells in MNCs depleted of CD38+ NK cells. [file 13075_2019_2001_MOESM12_ESM.doc]

**Table S7. The proportions of IL-10+ Treg cells in MNCs** depleted of CD38+ NK cells

| **Column** | **A** | **B** | **C** | **D** | **E** | **F** | **G** | **H** | **I** | **J** | **K** | **L** |
| --- | --- | --- | --- | --- | --- | --- | --- | --- | --- | --- | --- | --- |
| **IL-10+ Treg (%)** | 3.38±1.05 | 4.76±1.60 | 0.85±0.38 | 3.01±1.03 | 6.44±1.09 | 8.72±0.88 | 0.77±0.61 | 0.61±0.63 | 1.08±0.37 | 0.92±0.13 | 4.6±0.95 | 7.86±1.05 |
| **CD38+ NK** | - | - | + | + | + | + | + | + | + | + | + | + |
| **TNF-α** | - | - | - | - | + | + | - | - | - | - | - | - |
| **IFN-γ** | - | - | - | - | - | - | + | + | - | - | - | - |
| **anti-TNF-α Ab** | - | - | - | - | - | - | - | - | + | + |  |  |
| **anti-IFN-γ Ab** | - | - | - | - | - | - | - | - | - | - | + | + |
| **PBS** | + | - | + | - | + | - | + | - | + | - | + | - |
| **C3G** | - | + | - | + | - | + | - | + | - | + | - | + |
