# Supplementary material for: Potential therapeutic effects of cyanidin-3-O-glucoside on rheumatoid arthritis by relieving inhibition of CD38+ NK cells on Treg cell differentiation
Source: Arthritis Res Ther. 2019 Oct 28;21:220. doi: 10.1186/s13075-019-2001-0 (PMC6819496; doi:10.1186/s13075-019-2001-0)
Supplement: Supplementary file 13 — Additional file 13: Table S8. Lymphocyte subset proportion (%) in peripheral blood of CIA rats. [file 13075_2019_2001_MOESM13_ESM.doc]

**Table S8. Lymphocyte subset proportion (%) in peripheral blood of CIA rats**

|  | **Normal control** | **CIA control** | **CIA with Sirt6 inhibitor treatment** | **CIA with C3G treatment** | **CIA with C3G+Sirt6 inhibitor treatment** |
| --- | --- | --- | --- | --- | --- |
| **CD38+ NK** | 1.42±0.63 | 3.58±1.49 | 5.23±1.23 | 2.09±0.93 | 3.93±1.56 |
| **Treg** | 4.42±2.24 | 2.41±1.27 | 1.33±0.72 | 4.35±1.94 | 1.65±1.02 |
