# Supplementary material for: Potential therapeutic effects of cyanidin-3-O-glucoside on rheumatoid arthritis by relieving inhibition of CD38+ NK cells on Treg cell differentiation
Source: Arthritis Res Ther. 2019 Oct 28;21:220. doi: 10.1186/s13075-019-2001-0 (PMC6819496; doi:10.1186/s13075-019-2001-0)
Supplement: Supplementary file 14 — Additional file 14: Table S9. Lymphocyte subset proportion (%) in CIA synovial fluid. [file 13075_2019_2001_MOESM14_ESM.doc]

**Table S9. Lymphocyte subset proportion (%) in CIA synovial fluid**

|  | **CIA control** | **CIA with Sirt6 inhibitor treatment** | **CIA with C3G treatment** | **CIA with C3G+Sirt6 inhibitor treatment** |
| --- | --- | --- | --- | --- |
| **CD38+ NK** | 6.80±3.14 | 10.39±3.14 | 3.62±1.90 | 8.92±4.49 |
| **Treg** | 2.74±0.83 | 1.96±0.69 | 7.60±2.89 | 2.48±0.97 |
